# Reward-based option competition in human dorsal stream and transition from stochastic exploration to exploitation in continuous space

**DOI:** 10.1126/sciadv.adj2219

**Published:** 2024-02-23

**Authors:** Michael N. Hallquist, Kai Hwang, Beatriz Luna, Alexandre Y. Dombrovski

**Affiliations:** ^1^ Department of Psychology, University of North Carolina, Chapel Hill, NC, USA.; ^2^ Department of Psychological and Brain Sciences, Iowa Neuroscience Institute, University of Iowa, Iowa City, IA, USA.; ^3^ Department of Psychiatry, University of Pittsburgh, Pittsburgh, PA, USA.

## Abstract

Primates exploring and exploiting a continuous sensorimotor space rely on dynamic maps in the dorsal stream. Two complementary perspectives exist on how these maps encode rewards. Reinforcement learning models integrate rewards incrementally over time, efficiently resolving the exploration/exploitation dilemma. Working memory buffer models explain rapid plasticity of parietal maps but lack a plausible exploration/exploitation policy. The reinforcement learning model presented here unifies both accounts, enabling rapid, information-compressing map updates and efficient transition from exploration to exploitation. As predicted by our model, activity in human frontoparietal dorsal stream regions, but not in MT+, tracks the number of competing options, as preferred options are selectively maintained on the map, while spatiotemporally distant alternatives are compressed out. When valuable new options are uncovered, posterior β_1_/α oscillations desynchronize within 0.4 to 0.7 s, consistent with option encoding by competing β_1_-stabilized subpopulations. Together, outcomes matching locally cached reward representations rapidly update parietal maps, biasing choices toward often-sampled, rewarded options.

## Teaser

Information-compressing reinforcement learning updates value-laden maps in the human dorsal stream, facilitating exploitation.

## INTRODUCTION

Organisms face a difficult dilemma between exploiting options that are known to be good and exploring alternative options that might be even better. When a vertebrate faces a few discrete options, the striatum and amygdala can resolve the explore-exploit dilemma by representing options egocentrically (e.g., right/left) and tuning the exploration rate based on mesostriatal dopaminergic signals (*1*–*4*). In more complex terrestrial environments, however, quadrupeds rely on world-centric hippocampal cognitive maps that incorporate reinforcement (*5*–*7*). These mechanisms work efficiently at slower timescales; however, exploration and exploitation become more demanding when we move through a rapidly changing environment.

For example, primates adapted to arboreal hunting and foraging on terminal branches evolved visuomotor systems for fast and precise visually guided actions (*8*). The cortical “where” stream or the dorsal attention network (DAN) integrates visual and somatosensory information to build dynamic world-centric maps that guide visual search, locomotion, and grasp. The posterior parietal cortex (PPC) constructs maps using visual inputs from temporo-occipital areas such as MT+ as well as rostral parietal somatosensory inputs. In turn, the PPC sends map-based outputs to the frontal dorsal and ventral premotor (PMd and PMv) cortex and the frontal eye fields (FEFs) that guide action (*9*–*11*).

Visuomotor learning has to occur at a faster timescale than instrumental learning in the basal forebrain and striatum, which integrates reinforcement slowly and retains long-term values (*12*). Moreover, PPC maps contain rich visuomotor data necessary to decide what actions are likely to succeed in current and upcoming spatiotemporal locations, e.g., when an insect can be grasped on a moving branch (*13*–*15*). These pragmatic maps represent programs of movement toward currently available options that are based on prior visuomotor experience (*9*). Studies of gaze control, for example, find that PPC facilitates goal-congruent saccades by comparing what one is looking at versus what one is looking for (*10*).

How are these goals set? The PPC integrates past visuomotor experience and rewards (*16*), establishing bidirectional links between attention and learning (*15*). Visual stimuli repeatedly paired with rewards gradually gain priority on the PPC map and will be preferred in visuomotor interactions such as grasping actions. When a primate faces an array of potentially valuable options, PPC subpopulations representing them compete for behavioral selection (*9*). Yet, although PPC maps contain reward statistics, we lack a consensus account of how these statistics are learned.

Spatial working memory (WM) models using a buffer of recent outcomes explain rapid learning dynamics and the structure of PPC representations in deterministic environments (*17*, *18*). WM models, however, make no predictions about exploration versus exploitation and fail to explain long-term value or salience representations in PPC (*19*, *20*). The inability of the WM buffer to retain longer-term reinforcement history becomes a hindrance in stochastic environments, prompting researchers to invoke reinforcement learning (RL), at least as a parallel process (*21*). RL, on the other hand, tracks the options’ long-term values, which enables it to resolve the explore-exploit dilemma. Yet, learning dynamics in traditional RL models are often too slow to explain sensorimotor behavioral adaptation. Collins and colleagues have suggested that frontoparietal WM systems support rapid learning until the options become too many and need to be remembered for too long, at which point the mesocortical RL system takes over. This hybrid model explains how values of many discrete options may be learned. However, when a reward is obtained in an environment navigated by map (such as the sensorimotor space), values at the correct locations must be updated, presenting a credit assignment problem, which only a system containing the map can resolve. We also know that fast motor decisions are signaled in the dorsal cortex before mesostriatal inputs can be received and are instead guided by locally stored policies (*22*). In summary, existing models do not fully explain how rewards help resolve option between options encoded by dorsal stream maps.

**Fig. 1.**
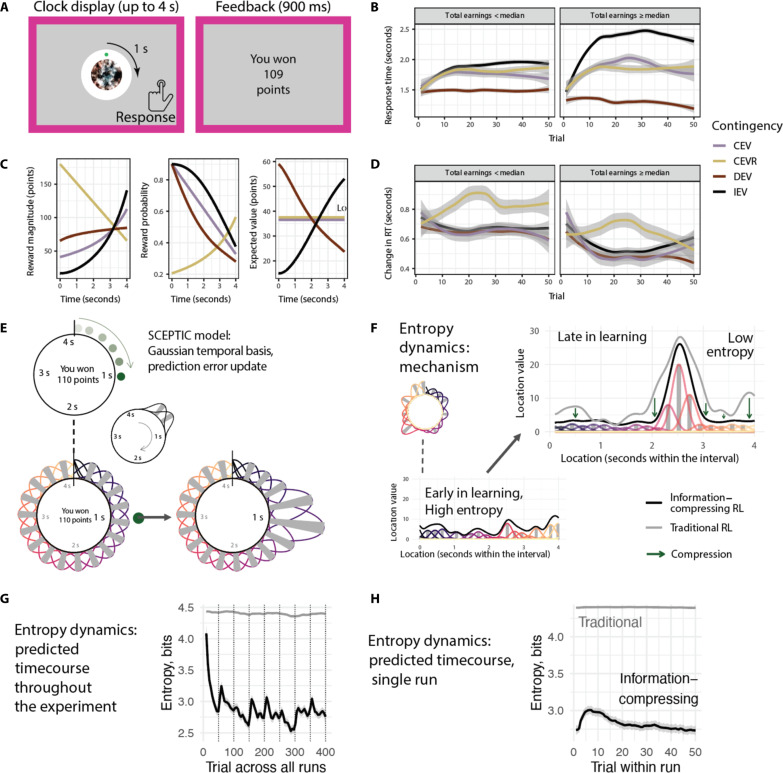
Paradigm and SCEPTIC model. (**A**) The clock paradigm consists of decision and feedback phases. During the decision phase, a dot revolves 360° around a central stimulus over the course of 4 s. Participants press a button to stop the revolution and receive a probabilistic outcome. (**B**) Evolution of subjects’ RTs by contingency and performance. Panels represent participants whose total earnings were above or below the sample median. (**C**) Rewards are drawn from one of four monotonically time-varying contingencies: two learnable (IEV and DEV, dark colors) and two unlearnable (CEV and CEVR, light colors). Reward probabilities and magnitudes vary independently. (**D**) Evolution of subjects’ RT swings by contingency and performance. (**E**) SCEPTIC model: Basis function representation. Top: Subject responds at 1 s and wins 110 points. Bottom left: The one-dimensional space of the task is tiled with Gaussian-shaped learning elements with staggered receptive fields. Bottom right: The reward at 1 s updates expected values (weights) of nearby basis elements. (**F**) Entropy dynamics of the information-compressing model, mechanism. Left: Example value distribution early in learning, when all locations have similar values and entropy is high (top: visual space of the task; bottom: linear coordinates with location on abscissa). Right: Example value distribution late in learning, contrasting information-compressing RL (black line, colored bases) with traditional RL (gray line). Information compression (green arrows) reduces the entropy of the value distribution. (**G**) Entropy dynamics of the information-compressing versus traditional RL model across the entire experiment. High initial entropy reflects random uniform prior basis element weights; however, qualitatively, the same dynamics are seen with priors of 0 (fig. S1, D to F). (**H**) Same as (G), for an average run. First run is excluded to eliminate effect of priors.

Here, we evaluate an alternative account of value-based option competition in the DAN: information-compressing RL, testing it against traditional RL with long-term value persistence and WM, alone or in combination. We experimentally manipulated the distribution of rewards during rapid movement through a one-dimensional continuous space (Fig. 1A), inducing value-laden visuomotor representations. Leveraging a validated computational model (*23*), we demonstrate that option competition in the DAN cannot be fully explained by WM or traditional RL and can be described by an information-compressing RL process that selectively maintains the values of preferred options and allows nonpreferred alternatives to decay. Selectively maintaining the values of preferred options reduces the entropy or compresses the information contained in the value function (*23*). Thus, our information-compressing RL model learns and forgets faster than RL without decay (termed “traditional RL” below), almost as quickly as WM models.

Moreover, whereas WM models fail to explain empirical findings of learned value or salience signals in the PPC (*19*, *24*), information-compressing RL accounts for them and makes a key neural prediction: Increases in the entropy of the learned value function, and consequently the number of potentially good options, should recruit more PPC neuronal subpopulations representing them. Entropy decreases should have the opposite effect, as fewer subpopulations dominate the output and behavior shifts from exploration to exploitation. Critically, traditional RL does not predict entropy decreases during successful learning and does not link entropy dynamics to exploitation (*23*). WM models, on the other hand, predict divergent entropy dynamics, determined only by the content of the buffer.

Biophysical models and electrophysiological studies of option competition in the posterior cortex suggest that each competing option may be encoded by a subpopulation with a unique phase of β_1_/α oscillatory output (*17*, *18*, *25*–*28*). Thus, we hypothesized that increases in the number of similarly valued options (i.e., entropy increases) would induce a β_1_/α desynchronization. We also examined whether blood-oxygen-level-dependent (BOLD) signal and oscillatory dynamics consistent with information-compressing RL predicted a successful explore-exploit transition. Two studies of DAN BOLD and one magnetoencephalography (MEG) study of posterior oscillations provided evidence supporting information-compressing RL.

## RESULTS

We begin by describing (i) behavior on the clock task and our model, SCEPTIC (strategic exploration/exploitation of instrumental contingencies), and (ii) the connectivity-based parcellation of the human DAN. Our main analyses focus on entropy dynamics and the transition from exploration to exploitation. We report on distinct neural substrates of exploration elsewhere.

### Behavior and SCEPTIC model

On the clock task (Fig. 1A), participants explore and learn reward contingencies within a 4-s time interval. A rotating dot marks the passage of time, reducing demands on internal timing. They are told to find the response time (RT) that yields the most points. Four stochastic reward contingencies with varying reward probability/magnitude trade-offs (Fig. 1C) encourage extensive exploration and trial-by-trial learning, requiring integration of reinforcement across trials and impeding purely WM-based or heuristic strategies. Whereas people’s responses shifted toward value maxima in learnable contingencies (Fig. 1B), participants almost never responded as early as possible in decreasing expected value (DEV) or as late as possible in increasing expected value (IEV). Thus, they did not recognize that contingencies were monotonic, instead searching for a subjective value maximum (RT_Vmax_); their estimate of its location often shifted within the block. Trial-wise changes of RTs (also known as “RT swings”) provide a model-free index of exploration. Early in learning, better-performing participants displayed large RT swings followed by a decline as they shifted to exploiting the subjective value maximum. Less successful participants kept exploring stochastically, with moderately large RT swings throughout, never settling on a clear value maximum. Curiously, successful participants transitioned from exploration to exploitation even in unlearnable contingencies where no objective value maximum exists [Fig. 1D, previously detailed in (*23*)]. As expected, compared to the functional magnetic resonance imaging (fMRI) session, performance was marginally better in the more comfortable MEG laboratory (table S1). As detailed in the next section, this behavior can be explained by adaptive selective maintenance of reinforcement histories.

SCEPTIC approximates the value function or expected reward by covering the space (interval) with a set of learning elements with staggered temporal receptive fields (*29*, *30*). Each element learns from temporally proximal rewards, updating its weight by reward prediction errors or the discrepancy between model-predicted reward at the chosen RT and the obtained reward (Fig. 1E). The highest-valued RT is the global maximum (also known as RT_Vmax_) of the model-estimated value function (Fig. 1F).

A key insight follows from our model: The explore-exploit balance depends on the entropy of the value function (*23*) [see policy entropy in artificial intelligence (*31*)]. Consider a learning agent who tracks the set of expected rewards or values associated with each target or option, termed the value function. When the estimated values of all options are equal, the entropy of the value function is highest, and the agent needs to explore to discover superior options. Conversely, when a single superior option (global value maximum) can be exploited, entropy is low. Thus, entropy of the value function quantifies global uncertainty about which option is best. In information theory terms, locations (approximated by basis elements) are an alphabet, reinforcement sequences are messages, and the value function is an information source encoding the reinforcement history. The information content of this source is given by Shannon’s entropy of the normalized value function (element weights), which is high when multiple attractive options compete and low when a single option dominates (Fig. 1F). Exploration generally increases the mutual information between the learned and objective value functions. However, a reward-maximizing agent only needs to discover the highest-valued options, rather than attempting to learn precisely the value of every option (*32*). Furthermore, maintaining and updating a detailed map incurs a high memory cost and a risk of cognitive failure, which humans strive to minimize (*33*). Thus, a resource-rational agent should reduce the entropy of its learned value function [see (*34*)].

In a previous report of behavior and computational modeling in this sample, we compared the information-compressing SCEPTIC selective maintenance model to several alternatives, including temporal difference learning, as well as models with uncertainty-directed exploration [here, we report on a subset of 70 participants who had usable fMRI data from the larger sample of *N* = 76 whose behavioral results were reported in (*23*)]. The selective maintenance was not only successful in simulated environments and afforded the best fit to human behavior on the clock task. While a model sensitive to local uncertainty was only second best, it indicated that instead of engaging in uncertainty-directed exploration, participants tended to avoid uncertain options in the large continuous space, likely due to cognitive constraints as our subsequent behavioral study suggests (*33*). Crucially, entropy dynamics captured by the selective maintenance model were strongly linked with successful exploration and exploitation: Subjects who earned more points tended to explore in early trials, increasing entropy, and converged on preferred options late in learning, decreasing entropy. This pattern was not observed using value entropy derived from the traditional RL variant with full maintenance, underscoring that the information-compressing dynamics of the selective maintenance model help resolve the explore-exploit dilemma.

Thus, our predictions about neural population activity corresponding to map updates were based on the information dynamics of the value function. Specifically, entropy increases signify that competing options emerge on the global map. We have shown that these information dynamics, barely detectable in traditional RL, become prominent when a resource-rational agent selectively remembers the values of favored actions and compresses out nonpreferred alternatives (Fig. 1, G and H, and fig. S1, D and E) (*23*). This compression heightens the relative dominance of the best actions, facilitating exploitation and exploration (fig. S1, A to C), scaling with performance and nonverbal intelligence (*23*). We tested the neural predictions of this information-compressing model against activity in the DAN, comparing it to otherwise identical traditional RL variant of SCEPTIC with long-term persistence of values and a representationally homologous WM model (for details, see Materials and Methods).

### Connectivity-based parcellation of the human DAN

**Fig. 2.**
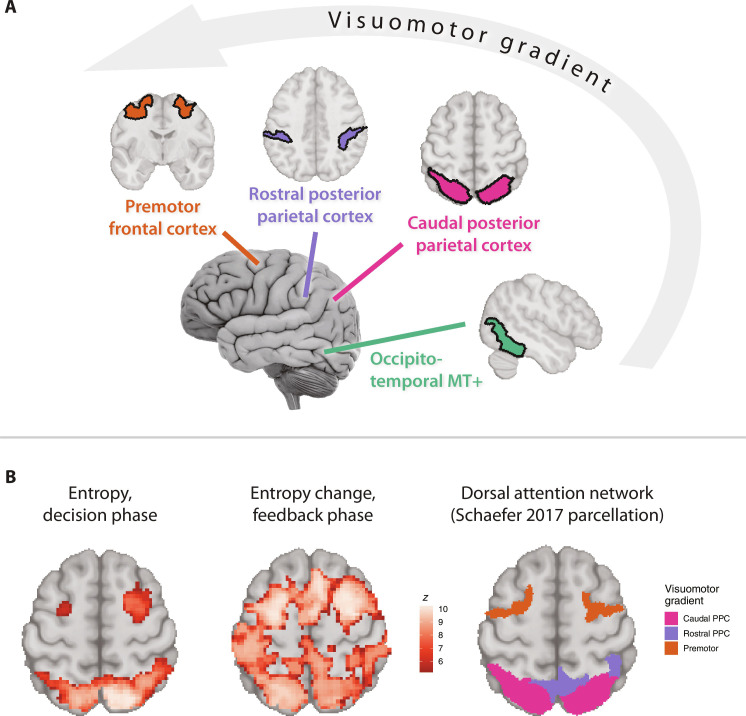
Human DAN and responses to value entropy and its change. (**A**) DAN nodes arranged along the visuomotor transformation gradient, connectivity-based parcellation of Schaefer *et al.* (*36*) (details: table S2; detailed parcellation: fig. S2). (**B**) Responses to value entropy (left) and entropy change (middle), voxelwise GLM; Schaefer DAN parcellation for the same axial slice (right; *z* = 55). Details: tables S3 and S4. Responses to reward/omission: fig. S3. We note that including entropy, entropy change, and prediction errors simultaneously in GLM analyses does not meaningfully change the pattern of results. This is due to the relatively low level of correlation among these signals.

Studies of functional connectivity in the human cortex identify a DAN (*35*–*38*), encompassing the temporo-occipital (putative human MT+), posterior parietal (IPS and SPL), and frontal premotor regions (FEF, PMv, and PMd). DAN is further subdivided into two subnetworks along the caudorostral visuomotor gradient: caudal, consisting of MT+ and caudal PPC, and rostral, consisting of rostral PPC and frontal premotor regions (Fig. 2) (*35*, *36*). Below, we describe activity across these four groups of regions. Because DAN subregions are characterized extensively in the macaque, for reference we label the human connectivity-based subregions according to their putative homology with monkey areas (*39*–*46*) (fig. S2 and table S2; we omit “putative human” from region names for simplicity noting that the interpretation of our results does not depend on precise homology with monkey areas).

### Value information dynamics in the dorsal stream and the transition from exploration to exploitation

While fMRI and MEG cannot access option representations in individual neurons or subpopulations, our analyses of learned value information dynamics enabled us to adjudicate among competing accounts based on population-level measures. Specifically, if a subpopulation is recruited to represent the value of each option, the number of active subpopulations should scale with the information content of the learned value function. Thus, we can test whether maps contained in the human DAN undergo reinforcement-based updates as predicted by our model by regressing trial-by-trial entropy change against BOLD signal and posterior oscillatory power (Fig. 1F). Entropy change reflects a global update to the dispersion of values for chosen and unchosen options and is distinct from prediction errors, which only reflect the local update to the chosen option, conceptually as well as statistically (|*r*| < 0.1 for signed or absolute prediction errors across both samples reported here). Moreover, the information-compressing versus traditional RL variants of the SCEPTIC model make different predictions about the nature of entropy change. Under the information-compressing model, entropy change has two components: (i) the decay of unchosen options, which reduces entropy, and (ii) value updates to the chosen action, which can increase or decrease entropy depending on whether the update promotes the dominant option relative to alternatives. By comparison, entropy change under the traditional RL model depends only on updates to the chosen action, and entropy is generally higher relative to the information-compressing model (*23*). To demonstrate information-compressing learning dynamics, we contrasted the neural fit of our information-compressing model with an otherwise identical traditional RL comparator lacking information compression. We further ascertained that observations were replicable and robust to potential confounds and individual heterogeneity.

#### Model-predicted value map information dynamics and DAN BOLD

Our whole-brain analysis revealed that the number of potentially advantageous options measured by model-predicted value entropy and its change (Fig. 2B and tables S3 and S4) recruited frontoparietal regions of the DAN but not MT+, the basal ganglia, or thalamus. Entropy change additionally recruited nodes of the cinguloopercular network (dorsal anterior cingulate cortex and anterior insula/frontal operculum) and the rostrolateral prefrontal cortex.

**Fig. 3.**
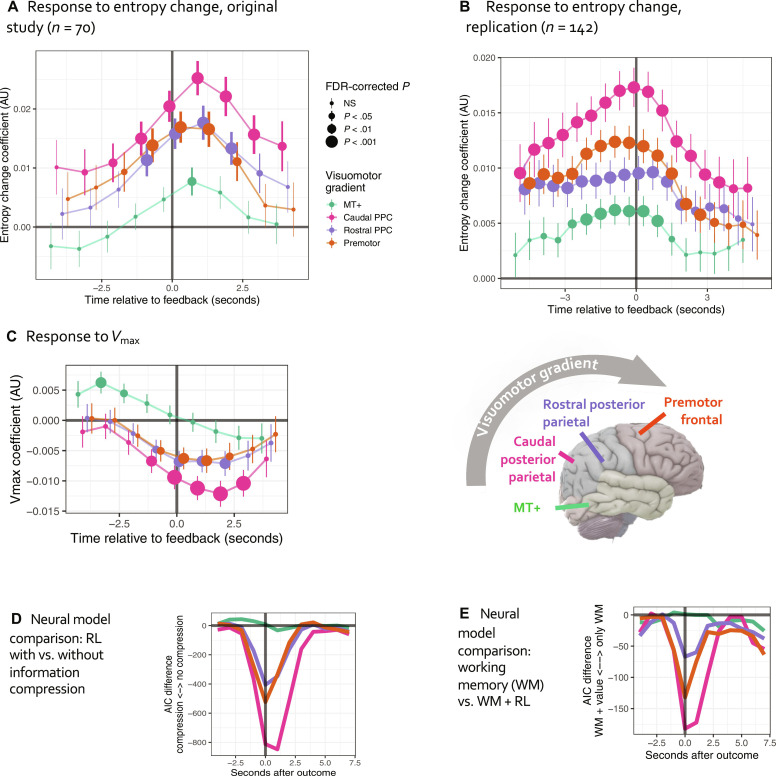
Information dynamics of the value function, DAN BOLD signal. (**A**) Responses to value entropy change (higher signal to increases reflecting a rising number of potentially valuable options), multilevel analysis of deconvolved BOLD signal from the original study, TR = 1 s. Error bars represent the SE of the estimate from the multilevel model. (**B**) Same, replication sample of older adults with and without depression, TR = 0.6 s. NB: Because BOLD response is smooth, we can only interpret the peak response as indicating the timing of activity. (**C**) Response to scalar *V*_max_, original study. (**D**) Neural model comparison providing evidence of information-compressing rather than traditional RL. (**E**) Analogous comparison demonstrating that DAN responses to entropy change cannot be explained solely in terms of spatial WM updates. AU, arbitrary units; NS, not significant.

If DAN responses correlated with entropy change reflect value map updates, then activity should be modulated after reinforcement. Analyses of within-trial BOLD activity revealed that entropy change modulated frontoparietal DAN activity after outcome, particularly in caudal PPC and frontal-premotor nodes (Fig. 3A); responses in MT+ were much weaker. In contrast, responses to scalar value of the best option (Fig. 3C) were positive in MT+ and negative in frontoparietal nodes. Effects of entropy change were evident with or without accounting for between-subject heterogeneity (individual random slopes), behavioral confounds (current and lagged RTs), and spatially nonspecific reinforcement features [scalar *V*_max_ (Fig. 3C), reward/omission, prediction error all included as covariates in Fig. 3A; model without covariates: fig. S4A].

Value entropy rises during early exploration when many options are sampled (Fig. 1H), and its association with neural responses could be an artifact of novelty. To rule out this confound, we manipulated value entropy by changing the reward contingency every 40 trials without any explicit cues in a follow-up study of 142 older individuals with and without psychopathology. In this replication study, frontoparietal DAN responses to entropy change were qualitatively unchanged, although we had excluded the first 10 trials from analyses to eliminate novelty effects (Fig. 3B). We further note that these analyses controlled for trial number to ensure that DAN responses reflected the average tendency across the experiment (see Materials and Methods for details).

#### Frontoparietal BOLD dynamics: Evidence of information compression

The long-term persistence of value is a hallmark of traditional instrumental learning. In contrast, our information-compressing model selectively maintains values of preferred regions, whereas values of spatiotemporally distant alternatives decay. To find evidence of such compression, we compared the neural fit of entropy change signals from the information-compressing model versus an otherwise identical model without compression. The information-compressing model better accounted for DAN responses to entropy change, particularly in PPC-caudal and frontal-premotor regions, but not in MT+ (AIC_information-compresssing_ − AIC_traditional_RL_: ≥−847; Fig. 3D). Compression in the SCEPTIC model occurs as part of the reinforcement-driven update (Eq. 7), and that is exactly when its advantage is greatest.

#### Information-compressing learning versus WM updates

Participants could perform the clock task by holding recent choices and outcomes in WM and repeating recently rewarded choices. Human choices, however, are not adequately explained by such a process. The SCEPTIC-derived RT_Vmax_ explained substantial variance in choices even after accounting for a five-trial buffer of choices and outcomes (fMRI session: *t* = 5.42, MEG session: *t* = 6.43, table S5; outcomes >4 trials back had no detectable impact on choice).

Neural responses to value entropy could also reflect updates to a spatial WM buffer. Although the above behavioral analyses speak to the contrary, it was important to rule out this alternative account using neural data. By definition, spatial WM contains the history of chosen locations (RTs) and corresponding outcomes. Thus, to model WM information content in a manner directly comparable with that of SCEPTIC, we encoded the selection history using the same representational structure (basis functions and spatial generalization gradient), adding a buffer of recent outcomes (detailed in Materials and Methods). We then predicted neural activity with the entropy and entropy change of the selection history and outcome history (reflecting WM buffer updates; fig. S5, B and C) with and without corresponding SCEPTIC signals. As in our main analysis, responses to value entropy change peaked ~1 s after outcome (fig. S5A), and model fit improved by ≥182 Akaike Information Criterion (AIC) points after adding SCEPTIC predictors (Fig. 3E; and after accounting for individual random slopes, ≥1726 points). Overall, while our analyses replicate common findings of spatial WM representations in the DAN and specifically PPC, they support parallel information-compressing updates of option values.

Similarly, one could also argue that value entropy dynamics and their neural correlates are epiphenomenal to preceding RT swings, whether they reflect strategic exploration or stochastic or even off-task responses (*47*). To rule out this possibility, we quantified a shift in the local distribution of choices as the summed Kullback-Leibler divergence (KLD; a metric of divergence between distributions) of RTs for trials *t* − 4, *t* − 3, and *t* − 2 from the local distribution of RTs of the preceding three trials. Higher values of this measure reflect a history of larger RT swings. With (Fig. 3A) or without (fig. S5A) this KLD measure as a covariate, the entropy change effects were qualitatively unchanged, confirming that our results reflect value map updates rather than selection history. On trials following larger RT swings, we observed lower online rostral PPC activity and weaker frontoparietal responses to feedback (fig. S5B), potentially indicating lapses in sensorimotor activity and attention (see also a similar analysis of MEG below).

#### DAN sensitivity to the number of potentially valuable options and exploitation

**Fig. 4.**
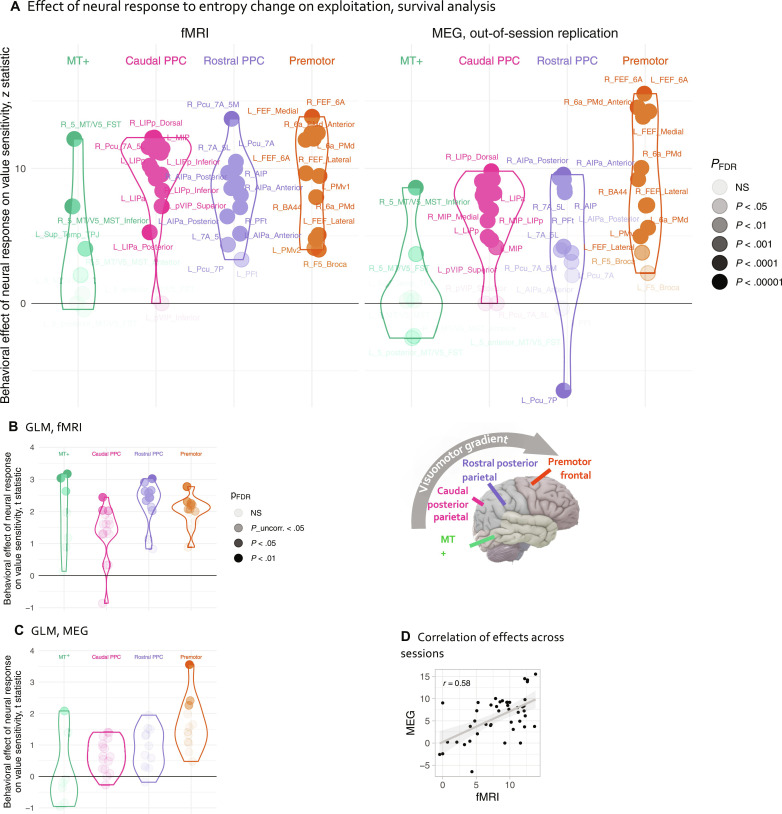
BOLD encoding of value information dynamics and behavioral exploitation. (**A**) Multilevel survival analyses examining how the individual’s neural response moderates their behavioral sensitivity to within-trial time-varying value. Left: Original fMRI session. Right: Replication, MEG session. Greater modulation of individual DAN BOLD response by value entropy change predicted more exploitative choices. (**B** and **C**) Same, GLM analysis. (**D**) The anatomical pattern of brain-behavior associations was preserved across the original fMRI and replication sessions. Each dot represents a single DAN parcel as labeled in (A).

The analyses above suggest that the DAN contains information-compressed value maps. Do these maps indeed govern the transition from exploration to exploitation? To answer this question, we tested whether individuals whose DAN activity better tracked with model-predicted entropy change made more value-sensitive, exploitative choices. We extracted entropy change regression coefficients (“betas”) for each DAN parcel from each subject, entering them as between-subjects predictors in a multilevel survival model predicting the momentary rate (hazard) of response with SCEPTIC-derived within-trial momentary value and its interaction with the fMRI beta. This interaction was positive across the parcels, indicating that individual value sensitivity scaled with entropy change responses across the DAN. This effect was replicated out of session (Fig. 4A; anatomical distribution of behavioral effect was preserved out of session Fig. 4D) and persisted after controlling for the nondecision time (censoring the first 1 s of the interval) and avoidance of missing the response window (censoring the last 0.5 s; fig. S6, E and F). With individual random slopes, effects were similar in the original sample, surviving false discovery rate (FDR) correction in the out-of-session replication only in premotor parcels (fig. S6, A and B). We ascertained that this effect did not depend on the Cox model proportional hazards assumption estimating an independent trial-level general linear model (GLM) predicting RTs with SCEPTIC-derived RT_Vmax_, the location of the highest-valued option, which enabled us to account for additional behavioral confounds (Materials and Methods, “fMRI analyses” section) and between-subject heterogeneity in value sensitivity (random value slopes; Fig. 4, B and C, and fig. S6).

#### *Value map information dynamics and posterior β*_1_/*α oscillations*

**Fig. 5.**
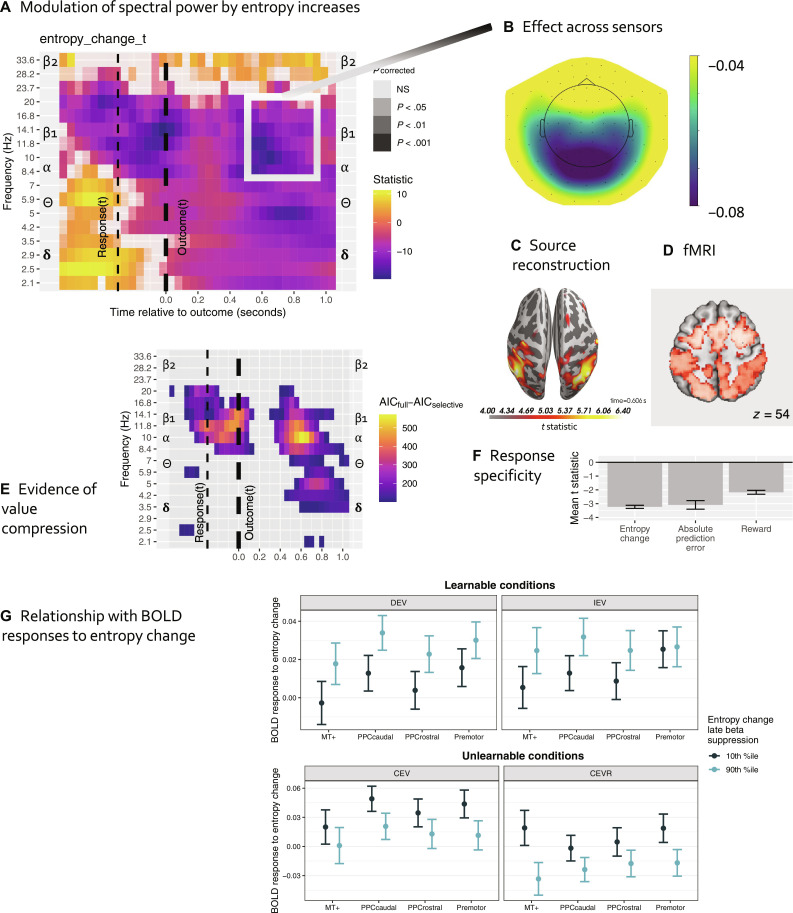
MEG: Oscillatory responses to value information dynamics and exploitation, anatomical and functional relationship to BOLD signal. (**A**) Oscillatory response to value entropy change: Cool colors represent desynchronization to increases reflecting a rising number of potentially valuable options, most prominent between 7 and 17 Hz (β_1_/α) band at 400 to 750 ms. (**B**) Neural model comparison providing evidence of information-compressing rather than traditional RL (see Fig. 3D). Hot colors represent AIC difference favoring the information-compressing (selective maintenance) model. (**C**) β_1_/α desynchronization was most evident in posterior sensors, consistent with a parietal source. (**D**) Source reconstruction localizes the β_1_/α suppression to the PPC. (**E**) fMRI BOLD map shown for comparison. (**F**) β_1_/α desynchronization was much better explained by value entropy change or absolute reward prediction errors than by reward/omission, model controlling for individual random slopes. More negative *t* statistics indicate a stronger effect. (**G**) Condition-level relationships between individuals’ BOLD and oscillatory responses to entropy change. Light blue bars represent individuals with stronger oscillatory responses (greater suppression to entropy increases). *Y* axis: higher coefficient values indicate stronger BOLD response. *X* axis: DAN regions from which BOLD response was extracted.

Having observed dynamic value maps encoded in frontoparietal DAN BOLD, we sought to understand cortical oscillation dynamics that underlie them. A late (550- to 1000-ms post-feedback) β_1_/α-band response has been reported on the clock task (*48*), but its functional role was unclear. We hypothesized that this response reflected an update to the parietal value map, with increases in the number of valuable options resulting in global desynchronization. Increases in entropy (and the number of valuable options) elicited suppression in the 7- to 17-Hz (β_1_/α) band at 400 to 750 ms, prominent in the posterior sensors (Fig. 5, A and B). The reconstructed sources of this signal followed an anatomical distribution similar to the pattern observed in fMRI (Fig. 5C versus Fig. 5D). As in our analyses of BOLD, late β_1_/α suppression was not explained by behavioral confounds (reward, RT*_t_*, RT_*t*−1_, and *V*_max_; Materials and Methods, “MEG analyses” section) and was strongly related to entropy change and absolute prediction errors, but not to reward/omission (Fig. 5F), suggesting that β_1_/α oscillations encode updates to the entire map of chosen and unchosen options, with the chosen option commanding additional processing. This β_1_/α response was evident in two learnable and one unlearnable condition. However, it was almost abolished in CEVR (constant expected value–reversed) [χ^2^(3) = 10.14, *P* < 0.018] where the probability/magnitude trade-off was the opposite of other conditions, indicating that the response was altered when outcomes did not match one’s expectations from previously encountered environments. This late suppression spread into the θ-band, peaking at 600 to 800 ms and 3 to 6 Hz, evident mostly in posterior sensors. In addition, an earlier burst of suppression at 8 to 17 Hz emerged immediately following response and ceased after the outcome (Fig. 5A), suggesting that participants were at times anticipating an increase in global uncertainty based on their response.

It is possible that, instead of β_1_/α desynchronization to entropy increases, our observations reflect β_1_/α synchronization to entropy decreases relative to baseline. We ruled out this possibility by separately examining the effects of entropy increase (versus decrease or no change) and entropy decrease (versus increase or no change; fig. S7, A and B). Whereas entropy increases elicited massive suppression at 8 to 20 Hz peaking at 0.4 to 0.8 s after outcome and spreading into the theta band, entropy decreases did not elicit synchronization of a similar magnitude.

One could also argue that effects of entropy increases merely reflect a recent history of highly variable choices (*47*) rather than updates to the distribution of learned value across competing options. While a recent history of RT swings measured by the Kullback-Leibler distance between RT_{*t*−3, *t*−2}_ and RT_*t*−1_ predicted suppression in the 7- to 16-Hz band (same model as above; fig. S7C), effects of entropy increases persisted while controlling for RT swings (fig. S7A), indicating that both selection and reinforcement history are encoded in β_1_/α oscillations. Last, we verified that our findings were robust to interindividual heterogeneity (*49*) by including the subject random slope of entropy change in our multilevel models (fig. S7D).

#### *Posterior* β*_1_/*α *oscillation dynamics: Evidence of information compression*

Our analyses of BOLD indicated that reinforcement representations in the frontoparietal DAN nodes were compressed as predicted by SCEPTIC. To understand whether similar information-compressing dynamics were reflected in oscillatory activity, we compared the fit of the multi-level model with entropy change regressor derived from either the information-compressing (exactly as in our main analysis above) or the traditional RL SCEPTIC model. The information-compressing model dominated in the 8- to 17-Hz band at 400 to 750 ms, in the 3- to 6-Hz band at 600 to 800 ms, and at 8- to 20-Hz peri-response (Fig. 5E), indicating that representations of competing options reflected in oscillatory activity displayed information-compressing dynamics predicted by the SCEPTIC model.

#### *Posterior β*_1_/*α oscillation dynamics and the explore-exploit transition*

To understand whether β_1_/α suppression responses to an increased number of options (entropy change) scaled with exploitation, we used models similar to those used in fMRI analyses (Fig. 4). To ensure that our results generalized across contingencies, we decomposed summary β_1_/α suppression responses into person-level means and condition-wise deviations. Person-level responses predicted exploitation [momentary value * β suppression response: *z* = 9.47, χ^2^(1) = 89.69, *P* < 10^−15^]. This effect was robust to between-subject heterogeneity [random slope of value, fixed effect: *z* = 2.14, χ^2^(1) = 4.57, *P* = 0.0326] and replicated out of session [*z* = 13.03, χ^2^(1) = 169.88, *P* < 10^−15^], even after accounting for between-subject variability in the effect of value [*z* = 3.40, χ^2^(1) = 11.60, *P* < 0.001]. After accounting for subject-level responses, no additional effect was observed, at the within-person, condition level [|*z*| ≤ 0.94, χ^2^(1) ≤ 0.86, *P* > 0.35], suggesting that the relationship of oscillatory responses and behavioral exploitation manifests at the between-person level.

#### *Magnitude of posterior β*_1_/*α response vis-à-vis BOLD*

Condition-level β_1_/α synchrony scaled negatively with BOLD responses across DAN, but only in learnable conditions, while the opposite pattern was seen in unlearnable conditions [Fig. 5G; β_1_/α response main effect: χ^2^(1) = 10.65, *P* = 0.0011, β_1_/α response * condition χ^2^(3) = 36.49, *P* < 10^−7^], suggesting that β_1_/α suppression and/or BOLD are differentially sensitive to the presence of an objective value maximum, and potentially also to the match between current and previously encountered contingencies.

## DISCUSSION

Our multimodal imaging study of human BOLD signal and cortical oscillations revealed that exploration and exploitation of a continuous sensorimotor space involves dynamic value maps in the dorsal stream. This map plasticity was not adequately explained by models of traditional RL or spatial WM but conformed to the predictions of an information-compressing RL model. More specifically, BOLD signals in the PPC and premotor cortex increased and posterior β_1_/α oscillations desynchronized in response to increases in the number of valuable options and, correspondingly, uncertainty about the best option. These BOLD and oscillatory dynamics predicted a successful behavioral transition from exploration to exploitation, replicating out of session and out of sample. BOLD dynamics consistent with map updates were seen throughout the parietal and frontal nodes of the dorsal stream but not in the occipitotemporal MT+.

Much debate about maps in the dorsal stream, especially in the PPC, has focused on what they represent: attentional priority (*50*–*53*), the intention to move (*54*), expected value versus salience of stimuli (*19*, *24*, *55*), or uncertainty and expected information gain (*56*–*60*). One explanation for this disagreement is that real-world motor programs are inextricable from visuospatial and value-laden representations of targets. This perspective aligns with the affordance competition account in which sensorimotor systems continuously encode opportunities for action emerging in the immediate environment (*9*, *61*). Affordance representations throughout the dorsal stream multiplex visual, oculomotor, motor, and somatosensory information (*62*–*64*). We observed that frontoparietal nodes along the visuomotor gradient from caudal PPC to premotor cortex respond similarly to the values of competing options, consistent with pragmatic, multimodal affordance representations. These dynamics were considerably weaker in the MT+, which instead responded to the recent reward and long-term value, potentially indicating that visuospatial processing was enhanced by higher reward rates [see (*65*)]. Thus, frontoparietal regions but not MT+ contain a spatially structured value map for all options.

Our results indicate that information processing in the dorsal stream is not exclusively feed-forward, as in traditional models of visuomotor transformation, but involves reinforcement-based feedback, addressing the critical question of how competition between multiple affordances is resolved (*9*, *66*, *67*). Cisek and colleagues conjectured that the prevailing affordance in output regions is determined by both RL and goals signaled to the dorsal stream by ventral prefrontal systems (*23*, *68*, *69*). We find that chosen and unchosen option values are updated on the PPC map within 0.4 to 0.7 s of reinforcement, predicting exploitation of the highest-valued option. Crucially, option value map updates and choices were better described by an information-compressing RL algorithm relative to traditional instrumental learning, even after accounting for updates to the visuospatial WM buffer (*70*). This algorithm selectively maintains preferred actions, compressing information about learned values and supporting more efficient exploitation during continuous visuomotor interactions. In general, RL algorithms will, to some extent, compress information contained in their value function if values of unchosen actions decay toward the floor of the value distribution. By definition, this will not be the case if unchosen values decay toward the mean of the distribution (*71*) or not at all (*60*). When an agent exploits successfully, a virtuous cycle emerges between higher-valued choices and decay of inferior options, increasing the degree of compression (*23*).

Dorsal stream regions are often characterized as “task positive” in fMRI studies, as their activity scales with the difficulty of the task. For example, in WM experiments, DAN regions including the PPC respond positively to the number of items to remember and top-down goals such as the selective maintenance of some stimuli (*72*, *73*). When choices are made under time pressure, PPC is recruited by the difference in expected value between alternative choices or choice difficulty (*74*). One can note the broad similarity between these effects and our findings, and it is hard to escape the conclusion that task difficulty can be expressed as the number of competing options or the information content of the task map. We cannot make strong claims as to what is being represented across various sensorimotor and cognitive tasks, particularly given that various representations, such as location and uncertainty [e.g., (*75*)], are often multiplexed. However, it is probable that reward-based information compression is a task-general process, which helps resolve option competition among subpopulations representing alternative affordances.

Although our results pertain to the role of the dorsal stream in the transition from stochastic exploration to exploitation, they also inform the interpretation of prior work on PPC involvement in exploration (*58*–*60*). For example, in a three-armed bandit task in which novel options periodically replaced familiar ones, Hogeveen and colleagues (*59*) found that peri-choice PPC activity scaled positively with the expected value of chosen option and negatively with its exploration bonus. However, novel (high-exploration bonus) and lower-valued options are more likely to be chosen when all options have similar values (i.e., when value function entropy is high). Thus, we cannot rule out the possibility—observed in our study—that PPC activity scaled with value function entropy and exploratory choices were stochastic rather than uncertainty-driven (at least not without explicitly controlling for value function entropy in analyses or manipulating it independently of the exploration bonus). The same logic can be applied to the findings of Daw and colleagues of higher PPC activity during exploratory choices (*59*). Likewise, Boorman and colleagues found that during the decision phase of a two-choice task, PPC activity was greater on trials in which participants decided to switch choices relative to the previous trial (*60*). These switches are by definition more likely when the entropy of the value function is high. Together, while our findings do not rule out PPC involvement in strategic exploration, future studies will need to explicitly dissociate incentives for strategic exploration from competition among close-valued options promoting stochastic exploration.

Our observations are not easily explained by a hybrid architecture considered in multi-armed bandit experiments wherein learning involves a combination of early (0.2 to 0.4 s) traditional RL updates and later (0.4 to 0.7 s) WM updates (*21*). During continuous sensorimotor interaction, we observe both value map and WM buffer updates at 0.4 to 0.7 s. How does the PPC obtain information about incoming reinforcement? The identity of a reward or goal state is probably cached in the dorsal and ventral stream (*69*), ensuring almost instantaneous reinforcement once the goal is reached (*68*). Alternatively, reinforcement can be signaled to the PPC by meso-striato-thalamo-cortical reward prediction errors. This feedback can be important at slower timescales but is unlikely to explain the posterior β_1_/α responses for several reasons. First, striatal signals that peak at 0.5 s for primary rewards (*76*) may not arrive early enough. Furthermore, considering the full cognitive architecture, visual monetary rewards must be first quantified in the PPC (*77*). The prior expected value at the precise location where the reward was harvested must be calculated using the PPC map. Only then can it be conveyed to the mesostriatal system, which would compute a prediction error and broadcast it through the basal ganglia output to the thalamocortical networks. Last, similar parietal responses at 0.4 to 0.7 s have been observed during perceptual learning in the absence of rewards or any other immediate feedback (*78*), suggesting that the rapid RL we describe is only one aspect of the more general process of parietal map updates. In this context, our results suggest that a dual-system account with competing frontoparietal WM and mesostriatal RL controllers (*21*) is not necessary to explain reward learning during sensorimotor interaction at subsecond timescales. In this case, affordance competition must largely be resolved through RL in the dorsal stream, with information compression facilitating a “within-system” decision (*79*).

**Fig. 6.**
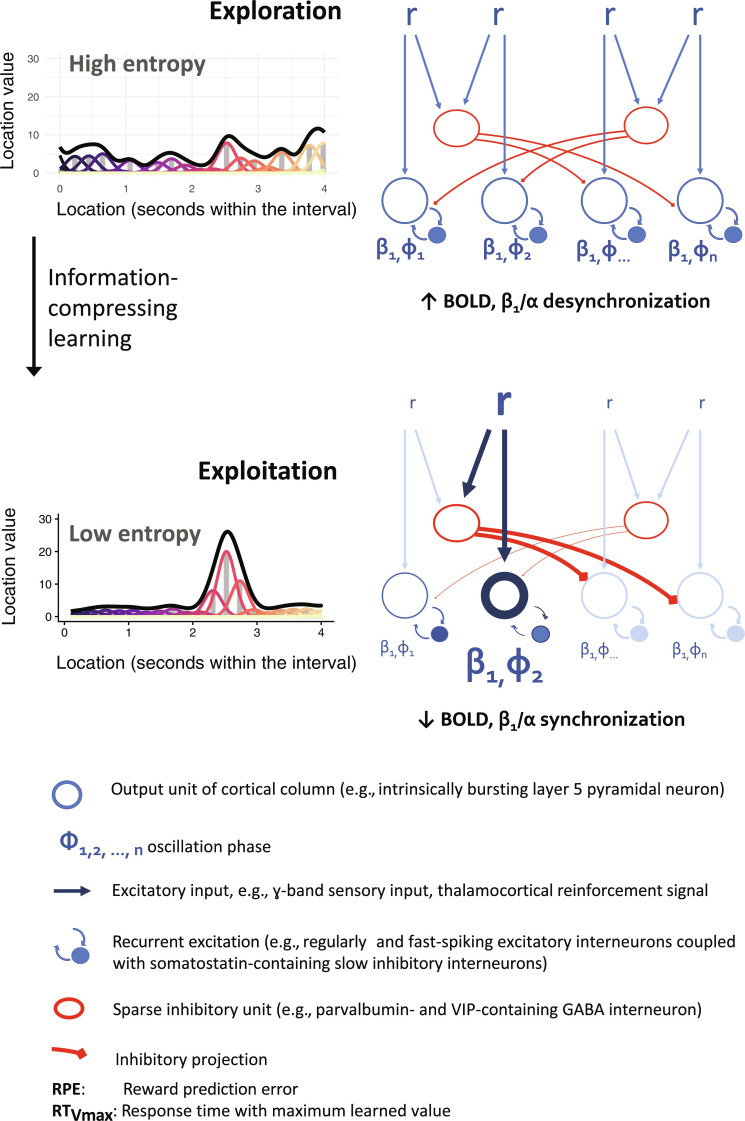
Option competition in the PPC: Conceptual model. Top: Exploration mode. Top left: Multiple options compete for selection, and the entropy of the value function is high. Top right: Competing options are represented by neuronal subpopulations with each producing oscillatory output with a distinct phase. Bottom: Exploitation mode. Top left: Following information-compressing learning, a global value maximum emerges. Top right: As a result of recurrent excitation and lateral inhibition, the subpopulation representing the dominant option begins to dominate the output. After Gelastopoulos, Whittington, and Koppel (biophysical model of β_1_/α-stabilized competing cortical populations) and Mysore and Kothari (computational models of competitive selection).

Parietal responses 0.4 to 0.5 s after stimulus have been linked to one-dimensional continuous map updates during perceptual learning (*78*). By mapping an ostensibly homologous value-laden map update to the β_1_/α band, we can bridge these observations with biophysically realistic circuit models. Gelastopoulos and colleagues (*27*) propose that competing representations in the parietal cortex are carried by β_1_/α-synchronized ensembles organized along cortical columns. It is thought that recurrent excitation stabilizes preferred option representations, while lateral inhibition suppresses nonpreferred alternatives (*27*, *80*, *81*). In line with these circuit-level models and empirical studies, we propose that during value-guided exploration of the sensorimotor space, recruitment of many ensembles produces an asynchronous oscillatory output, reflecting greater entropy of the value map and global uncertainty about the best action (Fig. 6) (*120*, *121*). When an option is preferentially sampled and reinforced, the β_1_/α-synchronized ensemble representing it comes to dominate the regional output, promoting exploitation. Compression of the value function may depend on lateral inhibition in which dominant ensembles suppress and even hijack the β_1_/α output of competing counterparts, reducing their likelihood of behavioral selection (*27*, *80*).

Dorsal stream BOLD was related to posterior β_1_/α desynchronization: Value entropy change modulated BOLD positively (Figs. 3, A and B, and 4, B and C) and β_1_/α power negatively (Fig. 5, A and E). BOLD and β_1_/α responses were correlated only in learnable contingencies. Moreover, oscillations were sensitive to a mismatch with previous experience: In a contingency with an unexpectedly reversed probability/magnitude trade-off, oscillatory responses no longer tracked entropy dynamics. While the spatial resolution of fMRI complements the temporal resolution of electrophysiology, BOLD and oscillatory power capture different aspects of cortical local field potentials. Their empirical correlations are generally positive in γ band and negative in α and β (which contain additional unique information about BOLD), with β but not α suppression accelerating increases and delaying decreases in BOLD (*80*–*82*). Thus, our fMRI and MEG findings align and ostensibly reflect updates to the dynamic value map. These functional properties were not shared by other frequency bands such as high β, θ, and δ.

Beyond simple visuomotor learning, what can the present observations tell us about more complex human decisions? Could the dorsal stream map plasticity that enabled our ancestors to forage on trees help us explore large abstract decision spaces and problem-solve under time pressure? For example, most studies of individual differences in human decision-making and their relation to psychopathology have used static discrete options and focused on basal forebrain-limbic circuitry and the prefrontal cortex. Yet, in a real-life crisis, decisions are often made during a sensorimotor interaction, under real or perceived time pressure, as one may receive an upsetting phone call or message and look around for cues as to what to do, which might lead them to the options of drinking or using drugs, or even a suicidal plan (*83*). We believe that individual differences in frontoparietal map plasticity—which show some measure of stability out of session (Fig. 4)—may in part explain disruptions of decision-making observed in personality disorders, addiction, and suicidal behavior.

The strengths of our study include consistent findings of value entropy dynamics in the human dorsal stream across MEG and fMRI modalities, across sessions, and in a separate fMRI sample. Experimental manipulation of reinforcement in a continuous space and our model provided access to a spatially structured value vector, dissociating global from local updates. Neural and behavioral computational model comparisons supported the inference that value maps in the dorsal stream are likely shaped by an information-compressing rather than traditional RL process or WM buffer updates. Our multilevel analyses revealed parallel within-trial temporal dynamics of cortical oscillations and deconvolved BOLD signals. Last, our observations of value entropy dynamics replicated in a modified experiment with unsignaled reversals, ruling out novelty as an alternative explanation.

The main limitation of our study is the lack of a causal manipulation that would isolate contributions of various DAN nodes to resolving the explore-exploit dilemma, although our findings of dynamic value maps in the dorsal stream are in line with human transcranial stimulation studies (*55*, *84*). Human neural stimulation and rodent optogenetic studies are needed to test our model of value-dependent affordance competition. Furthermore, we did not separately manipulate modality-specific sensory or motor demands, and future studies will need to investigate how exactly the reinforcement history modulates rapidly evolving multimodal sensorimotor option representations in the dorsal cortex as they guide reach, grasp, and make eye movements. It is also difficult to know whether our findings generalize to nontemporal spaces and to punishments as opposed to rewards. Last, we collected MEG and fMRI data in separate sessions, enabling out-of-session replication, but precluding an analysis of simultaneous BOLD and cortical oscillation recordings.

In conclusion, exploration and exploitation of a continuous sensorimotor space depend on dynamic value maps in the dorsal stream, particularly in the PPC. The dorsal stream selectively maintains values of preferred options and compresses out inferior, spatiotemporally distant alternatives, and value-based option competition is reflected in the β_1_/α oscillatory output of posterior cortical subpopulations. Option values in PPC persist beyond the timescale of the WM buffer. Our results support the affordance competition view of dorsal cortex maps and are at odds with the notion that sensorimotor choices involve a sequence of temporally distinct sensory, reward, cognitive, and motor computations. Together, our study sheds light on how competition between dorsal stream neuronal subpopulations may enable primates, including humans, to choose among multiple options in rapidly changing environments.

## MATERIALS AND METHODS

### Participants

Participants in the original study were 70 typically developing adolescents and young adults aged 14 to 30 (M = 21.4, SD = 5.1). Thirty-seven (52.8%) participants were female and 33 were male. Before enrollment, participants were interviewed to verify that they had no history of neurological disorder, brain injury, pervasive developmental disorder, or psychiatric disorder (in self or first-degree relatives). Participants in the replication study were 143 middle-aged and older adults aged 50 to 80 (M = 62.2, SD = 6.8), 80 (56%) were female and 62 were male; 101 were diagnosed with DSM-IV (the Diagnostic and Statistical Manual of Mental Disorders, Fourth Edition) nonpsychotic major depression. Individuals with a history of psychosis, mania, neurological conditions of the brain, and current substance use disorders were excluded from the replication study. Participants and/or their legal guardians provided informed consent or assent before participation in both studies. Experimental procedures for this study complied with the Code of Ethics of the World Medical Association (1964 Declaration of Helsinki) and the Institutional Review Board at the University of Pittsburgh (protocols PRO10090478 and STUDY19030288). Participants were compensated $75 for completing the original experiment and $150 for completing the replication study, which included other experiments.

### Procedure

#### Original study

As part of a larger study, participants completed an exploration and learning task (“clock task”; Fig. 1A and detailed below) in separate MEG and fMRI sessions. The order of the fMRI and MEG sessions was counterbalanced (fMRI first *n* = 34, MEG first *n* = 36), and the sessions were separated by 3.71 weeks on average (SD = 1.59 weeks).

During the fMRI session, participants completed eight runs of the clock task [based on (*85*)]. Runs consisted of 50 trials in which a green dot revolved 360° around a central stimulus over the course of 4 s. Participants pressed a button to stop the dot, which ended the trial. They then received a probabilistic reward for the chosen RT according to one of four time-varying contingencies, two learnable (increasing and decreasing expected value) and two unlearnable. All contingencies were monotonic but featured reward probability/magnitude trade-offs that made learning difficult [see (*23*) for more detailed analyses of the task]. After each response, participants saw the probabilistic reward feedback for 0.9 s. If participants failed to response within 4 s, they received zero points.

The central stimulus was a face with a happy expression or fearful expression or a phase- scrambled version of face images intended to produce an abstract visual stimulus with equal luminance and coloration. Faces were selected from the NimStim database (*86*). All four contingencies were collected with scrambled images, whereas only IEV and DEV were also collected with happy and fearful faces. The effects of the emotion manipulation will be reported in a separate manuscript because they are not central for the examination of the neural substrates of exploration and exploitation on this task.

Each trial was followed by an intertrial interval (ITI) that varied in length according to an exponential distribution. To maximize fMRI detection power, the sequence and distribution ITIs were derived using a Monte Carlo approach implemented by the optseq2 command in FreeSurfer 5.3. More specifically, we simulated 5 million possible ITI sequences consisting of 50 trials each and retained the top 320 orders based on their estimation efficiency. For each subject, the experiment software randomly sampled eight of these efficient ITI sequences, which were used for the durations of ITIs in the task.

During the MEG session, participants completed eight runs of the same task. The contingencies and trial structure were identical to fMRI (see Fig. 1A), requiring participants to respond within a 4-s interval to maximize the points they earned. Given the lower signal-to-noise ratio of MEG relative to fMRI, runs consisted of 63 trials each.

As detailed in the results, the behavioral data from the MEG and fMRI sessions were used to test the out-of-session consistency of brain-behavior effects identified by each modality. This enabled us to establish whether individual differences in dorsal stream activity and exploration/exploitation represented stable tendencies versus patterns incidental to a single experimental session.

#### Replication study

Procedures of the replication study were similar, but no MEG data were collected. Participants completed 240 trials of the clock task in two runs. Only IEV and DEV contingencies were used. To dissociate value entropy from novelty, the contingency reversed every 40 trials unbeknown to the participants. Trials were extended to 5 s to accommodate slower psychomotor speed in this older sample.

### Imaging acquisition and processing methods

#### fMRI acquisition

Neuroimaging data during the clock task were acquired in a Siemens Tim Trio 3T scanner for the original study and Siemens Tim Prisma 3T scanner for the replication study at the Magnetic Resonance Research Center, University of Pittsburgh. Because of participant-dependent variation in RTs on the task, each fMRI run varied in length from 3.15 to 5.87 min (M = 4.57 min, SD = 0.52). Functional imaging data for the original/replication study were acquired using a simultaneous multislice sequence sensitive to BOLD contrast, TR = 1.0/0.6 s, TE = 30/27 ms, flip angle = 55/45°, multiband acceleration factor = 5/5, voxel size = 2.3/3.1 mm^3^. We also obtained a sagittal MPRAGE T1-weighted scan, voxel size = 1/1 mm^3^, TR = 2.2/2.3 s, TE = 3.58/3.35 ms, GRAPPA 2/2× acceleration. The anatomical scan was used for coregistration and nonlinear transformation to functional and stereotaxic templates. We also acquired gradient echo fieldmap images (TEs = 4.93/4.47 ms and 7.39/6.93 ms) for each subject to mitigate inhomogeneity-related distortions in the fMRI data.

#### Preprocessing of fMRI data

Anatomical scans were registered to the MNI152 template (*87*) using both affine (ANTS SyN) and nonlinear (FSL FNIRT) transformations. Functional images were preprocessed using tools from NiPy (*88*), AFNI (version 19.0.26) (*89*), and the FMRIB software library (FSL version 6.0.1) (*90*). First, slice timing and motion coregistration were performed simultaneously using a four-dimensional registration algorithm implemented in NiPy (*91*). Nonbrain voxels were removed from functional images by masking voxels with low intensity and by the ROBEX brain extraction algorithm (*92*). We reduced distortion due to susceptibility artifacts using fieldmap correction implemented in FSL FUGUE.

Participants’ functional images were aligned to their anatomical scan using the white matter segmentation of each image and a boundary-based registration algorithm (*93*), augmented by fieldmap unwarping coefficients. Given the low contrast between gray and white matter in echoplanar scans with fast repetition times, we first aligned functional scans to a single-band fMRI reference image with better contrast. The reference image was acquired using the same scanning parameters but without multiband acceleration. Functional scans were then warped into MNI152 template space (2.3-mm output resolution) in one step using the concatenation of functional-reference, fieldmap unwarping, reference-structural, and structural-MNI152 transforms. Images were spatially smoothed using a 5-mm full width at half maximum kernel using a nonlinear smoother implemented in FSL SUSAN. To reduce head motion artifacts, we then conducted an independent component analysis for each run using FSL MELODIC. The spatiotemporal components were then passed to a classification algorithm, ICA-AROMA, validated to identify and remove motion-related artifacts (*94*). Components identified as noise were regressed out of the data using FSL regfilt (nonaggressive regression approach). ICA-AROMA has performed very well in head-to-head comparisons of alternative strategies for reducing head motion artifacts (*95*). We then applied a 0.008-Hz temporal high-pass filter to remove slow-frequency signal changes (*96*); the same filter was applied to all regressors in GLM analyses. Last, we renormalized each voxel time series to have a mean of 100 to provide similar scaling of voxelwise regression coefficients across runs and participants.

#### Treatment of head motion

In addition to mitigating head motion–related artifacts using ICA-AROMA, we excluded runs in which more than 10% of volumes had a framewise displacement of 0.9 mm or greater, as well as runs in which head movement exceeded 5 mm at any point in the acquisition. This led to the exclusion of 11 runs total, yielding 549 total usable runs across participants. Furthermore, in voxelwise GLMs, we included the mean time series from deep cerebral white matter and the ventricles, as well as first derivatives of these signals, as confound regressors (*95*).

#### MEG data acquisition

MEG data were acquired using an Elekta Neuromag VectorView MEG system (Elekta Oy, Helsinki, Finland) in a three-layer magnetically shielded room. The system consisted of 306 sensors, with 204 planar gradiometers and 102 magnetometers. In this project, we only included data from the gradiometers, as data from magnetometers added noise and had a different amplitude scale. MEG data were recorded continuously with a sampling rate of 1000 Hz. We measured head position relative to the MEG sensors throughout the recording period using four continuous head position indicators that continuously emit sinusoidal signals, and head movements were corrected offline during preprocessing. To monitor saccades and eye blinks, we used two bipolar electrode pairs to record vertical and horizontal electrooculogram (EOG).

#### Preprocessing of MEG data

Flat or noisy channels were identified with manual inspections, and all data preprocessed using the temporal signal space separation (TSSS) method (*97*, *98*). TSSS suppresses environmental artifacts from outside the MEG helmet and performs head movement correction by aligning sensor-level data to a common reference (*99*). This realignment allowed sensor-level data to be pooled across subject group analyses of sensor-space data. Cardiac and ocular artifacts were then removed using an independent component analysis by decomposing MEG sensor data into independent components (ICs) using the infomax algorithm (*100*). Each IC was then correlated with electrocardiogram (ECG) and EOG recordings, and an IC was designated as an artifact if the absolute value of the correlation was at least 3 SDs higher than the mean of all correlations. The non-artifact ICs were projected back to the sensor space to reconstruct the signals for analysis. After preprocessing, data were epoched to the onset of feedback, with a window from −0.7 to 1.0 s. Trials with gradiometer peak-to-peak amplitudes exceeding 3000 fT/cm were excluded. For each sensor, we computed the time-frequency decomposition of activity on each trial by convolving time-domain signals with Morlet wavelet, stepping from 2 to 40 Hz in logarithmic scale using six-wavelet cycles. This yielded trial-level time-frequency data that were amenable to multilevel models across frequencies and peri-feedback times.

### Computational model of behavior

#### Core architecture of SCEPTIC RL model

The SCEPTIC model represents the one-dimensional space/time of the clock task using a set of unnormalized Gaussian radial basis functions (RBFs) spaced evenly over an interval *T* in which each function has a temporal receptive field with a mean and variance defining its point of maximal sensitivity and the range of times to which it is sensitive, respectively (a conceptual depiction of the model is provided in Fig. 1). The primary quantity tracked by the basis is the expected value of a given choice (RT; we use the intuitive term value for continuity with prior studies of PPC maps; however, because this estimate does not converge on the true reward rate, it is technically a preference, see text following Eq. 7). To represent time-varying value, the heights of the basis functions are scaled according to a set of *B* weights, **w** = [*w*_1_, *w*_2_, …, *w_b_*]. The contribution of each basis function to the integrated value representation depends on its temporal receptive field

$$\varphi_{b}\left(x\right)=\text{exp}\left[-\frac{{\left(x-\mu_{b}\right)}^{2}}{2s_{b}^{2}}\right]$$ (1)

where *x* is an arbitrary point within the time interval *T*, μ*_b_* is the center (mean) of the RBF, and $s_{b}^{2}$ is its variance. In addition, more generally, the temporally varying expected value function on a trial *i* is obtained by the multiplication of the weights with the basis

$$V\left(i\right)=\sum_{b=1}^{B}w_{b}\left(i\right)\varphi_{b}$$ (2)

For the clock task, where the probability and magnitude of rewards varied over the course of 4-s trials, we spaced the centers of 24 Gaussian RBFs evenly across the discrete interval and chose a fixed width, $s_{b}^{2}$, to define the temporal variance (width) of each basis function. More specifically, $s_{b}^{2}$ was chosen such that the distribution of adjacent RBFs overlapped by approximately 50% [for details and consideration of alternatives, see (*23*)]. The basic model, referred to as traditional RL in Results, learns the expected values of different RTs by updating each basis function *b* according to the equation

$$w_{b}\left(i+1\right)=w_{b}\left(i\right)+e_{b}\left(i|t\right)\alpha \left[\text{reward}\left(i|t\right)-w_{b}\left(i\right)\right]$$ (3)

where *i* is the current trial in the task, *t* is the observed RT, and reward (*i* ∣ *t*) is the reinforcement obtained on trial *i* given the choice *t*. Prediction error updates are weighted by the learning rate α and the temporal generalization function or eligibility *e*. To avoid tracking separate value estimates for each possible moment, feedback obtained at a given RT *t* is propagated to adjacent times. Thus, to implement temporal generalization of expected value updates, we used a Gaussian RBF centered on the RT *t,* having width $s_{g}^{2}$. The eligibility *e_b_* of a basis function φ*_b_* to be updated by prediction error is defined as its overlap with the temporal generalization function, *g*

$$g\left(x\right)=\text{exp}\left[-\frac{{\left(x-t\right)}^{2}}{2s_{g}^{2}}\right]$$ (4)

$$e_{b}\left(i|t\right)=\int_{0}^{T}\text{min}\left[g\left(\tau \right),\varphi_{b}\left(\tau \right)\right]d\tau$$ (5)

where τ represents an arbitrary time point along the interval *T*. Thus, for each RBF *b*, a scalar eligibility *e_b_* between 0 and 1 represents the proportion of overlap between the temporal generalization function and the receptive field of the RBF. In the case of complete overlap, where the RT is perfectly centered on a given basis function, *e_b_* will reach unity, resulting in a maximal weight update according to the learning rule above. Conversely, if there is no overlap between an RBF and the temporal generalization function, *e_b_* will be 0 and that RBF will receive no update. For the eligibility to be bounded on the interval [0, 1], the basis functions are each normalized to have an area under the curve of unity (i.e., representing probability density). Here, we also fixed the width of the generalization function to match the basis (i.e., $s_{g}^{2}$ = $s_{b}^{2}$).

The SCEPTIC model selects an action based on a softmax choice rule, analogous to simpler RL problems [e.g., two-armed bandit tasks (*101*)]. For computational speed, we arbitrarily discretized the interval into 100-ms time bins such that the agent selected among 40 potential responses (i.e., a multinomial representation). At trial *i*, the agent chooses an RT in proportion to its expected value

$$p\left[t\left(i+1\right)=j|V\left(i\right)\right]=\frac{\text{exp}\left[V{\left(i\right)}_{j}/\beta \right]}{\sum_{\tau =0}^{T}\text{exp}\left[V{\left(i\right)}_{\tau }/\beta \right]}$$ (6)

where *j* is a specific RT and the temperature parameter β controls value sensitivity such that choices become more stochastic and less value sensitive at higher β values.

#### Information-compressing RL with selective maintenance

As detailed previously (*34*), a model that selectively maintained frequently chosen, preferred actions far outperformed alternative models. Specifically, basis weights corresponding to nonpreferred, spatiotemporally distant actions revert toward a prior in inverse proportion to the temporal generalization function

$$\begin{array}{c} w_{b}\left(i+1\right)=w_{b}\left(i\right)+e_{b}\left(i|t\right)\alpha \left[\text{reward}\left(i|t\right)-w_{b}\left(i\right)\right]-\\ \gamma \left[1-e_{b}\left(i|t\right)\right]\left[w_{b}\left(i\right)-h\right] \end{array}$$ (7)

where γ is a selective maintenance parameter between 0 and 1 that scales the degree of reversion toward a point *h*, which is taken to be 0 here for parsimony, but could be replaced with an alternative prior expectation. Our primary fMRI analyses used signals derived from fitting the information-compressing RL model (Eq. 7) to participants’ behavior, while comparisons with traditional RL used the model with the learning rule described in Eq. 3. Two features supported by computational studies and tests against human behavior (*23*), (i) the decay in the weights of unchosen alternatives and (ii) calculation of prediction errors based on individual element weights *w_b_* (Eqs. 3 and 7) rather than the estimated value function *V*(*i*) (Eq. 2), preclude *w_b_* or *V*(*i*) from converging on the true reward rate. While we refer to *V*(*i*) as expected value for continuity with previous studies of the parietal cortex (*19*, *24*, *55*), *w_b_* are closer to preferences in policy gradient algorithms (*32*, *102*, *103*). Here, we focus on testing the hypothesis of information compression (Eq. 8) and make no strong claims about whether representations of reinforcement in PPC constitute expected values or preferences. Although taken together with our earlier behavioral and computational results, neural model comparisons reported here can be taken to favor the preferences hypothesis, a definitive adjudication will require new experiments. Value versus policy learning accounts are not necessarily mutually exclusive since actor-critic algorithms combine both approaches.

As detailed in Results, we defined the information content of the learned value distribution as Shannon’s entropy of the normalized basis weights (the trial index *i* is omitted for simplicity of notation, and tilde denotes normalization)

$$H\left(\tilde{w}\right)=-\sum_{b=1}^{B}{\tilde{w}}_{b}{\text{log}}_{10}\left({\tilde{w}}_{b}\right)$$ (8)

Weights are normalized here only to calculate entropy, but not within the learning rule (Eq. 7). We further sought to examine whether entropy responses in the DAN were consistent with the information-compressing selective maintenance model. To test the specificity of the representation, we conducted analyses using entropy calculated from the information-compressing SCEPTIC selective maintenance model (Eq. 7) versus entropy from a traditional RL, full-maintenance counterpart that did not decay the values of unchosen actions [Eq. 3; detailed model comparisons provided in (*23*)].

#### WM model

We argue that information dynamics attributable to value entropy from the SCEPTIC information-compressing model best explain the exploration-exploitation transition in behavior and updates to the value map in the DAN. Yet, one could imagine that the DAN relies solely on a spatial WM representation with a buffer containing locations and outcomes of recent choices. In turn, the information content of this buffer might be sufficient to explain DAN BOLD activity.

We first examined whether a WM process alone is sufficient to explain human choices without invoking information-compressing RL. We used a multilevel regression model as described below (“Brain-behavior fMRI analyses using regression coefficients from model-based fMRI GLM analyses” section) to predict the participant’s current RT with *k* preceding RTs representing the selection history buffer and their interactions with reward/omission representing the outcome buffer. This analysis revealed no effect of outcomes beyond four trials (table S4). To assess the effect of the more remote reinforcement history not captured by the last *k* choices and outcomes, we then tested the incremental contribution of the RT_Vmax_ (time of the learned value maximum) derived from the SCEPTIC model.

To conduct a conclusive test of this alternative account, we created a strong WM-only comparator model that adopted the SCEPTIC RBF representation, using the basis weights to store the buffer of recently chosen locations, alongside a separate vector of recent outcomes. Specifically, the model remembered the past *k* choices by placing a unit-height eligibility functions at these locations and taking the sum, forming a selection history function, *s*(*x*). *k* was empirically estimated as 4 using multilevel linear regression (table S4). In turn, this representation of selection history was combined with the RBF by multiplying the selection history function and basis, yielding WM basis weights $w_{b}^{\text{WM}}$ whose height scaled with the selection history

$$s\left(x\right)=\sum_{j=i-5}^{i-1}\text{exp}\left\{-\frac{{\left[x-t\left(j\right)\right]}^{2}}{2s_{g}^{2}}\right\}$$ (9)

$$w_{b}^{\text{WM}}\left(i\right)=\int_{0}^{T}\left[s\left(\tau \right)\varphi_{b}\left(\tau \right)\right]d\tau$$ (10)

where *i* represents the current trial and *t*(*j*) is the RT on the *i*th previous trial. In turn, entropy can be calculated on the selection history basis weights in the same fashion as in the regular SCEPTIC model (Eq. 8). Outcome history was simply represented by a vector **o** containing 1 s for rewards and 0 s for reward omissions in the past four trials. This implementation did not require estimating free parameters from behavior. Thus, total entropy or information content of WM *H*^WM^ is the joint entropy of the normalized selection and outcome buffers (with tilde denoting normalization)

$$H_{\text{total}}^{\text{WM}}=H\left(\tilde{w}\right)+H\left(\tilde{o}\right)$$ (11)

#### Fitting of SCEPTIC model to behavioral data

SCEPTIC model parameters were fitted to individual choices using an empirical Bayesian version of the variational Bayesian approach (*104*). The empirical Bayes approach relied on a mixed-effects model in which individual-level parameters were assumed to be sampled from a normally distributed population. The group’s summary statistics, in turn, were inferred from individual-level posterior parameter estimates using an iterative variational Bayes algorithm that alternates between estimating the population parameters and the individual subject parameters. Over algorithm iterations, individual-level priors are shrunk toward the inferred parent population distribution, as in standard multilevel regression. Furthermore, to reduce the possibility that individual differences in voxelwise estimates from model-based fMRI analyses reflected differences in the scaling of SCEPTIC parameters, we refit the SCEPTIC model to participant data at the group mean parameter values. This approach supports comparisons of regression coefficients across subjects and reduces the confounding of brain-behavior analyses by the individual fits of the computational model to a participant’s behavior. We note, however, that our results were qualitatively the same when model parameters were free to vary across people (additional details available from the corresponding author upon request).

### fMRI analyses

#### Voxelwise fMRI GLM analyses

Voxelwise GLM analyses of fMRI data were performed using FSL version 6.0.4 (*90*). Single-run analyses were conducted using FSL FILM, which implements an enhanced version of the GLM that corrects for temporal autocorrelation by prewhitening voxelwise time series and regressors in the design matrix (*96*). For each design effect, we convolved a duration-modulated unit-height boxcar regressor with a canonical double-gamma hemodynamic response function (HRF) to yield the model-predicted BOLD response. All models included convolved regressors for the clock and feedback phases of the task.

Moreover, GLM analyses included parametric regressors derived from SCEPTIC. For each whole-brain analysis, we added a single model-based regressor from SCEPTIC alongside the clock and feedback regressors. Results were qualitatively unchanged, however, when all SCEPTIC signals were included as simultaneous predictors, given the relatively low correlation among these signals. For each model-based regressor, the SCEPTIC-derived signal was mean-centered before convolution with the HRF. The reward prediction error and entropy change signals were aligned with the feedback, whereas entropy was aligned with the clock (decision) phase. Furthermore, for regressors aligned with the clock phase, which varied in duration, we sought to unconfound the height of the predicted BOLD response due to decision time from the parametric influence of the SCEPTIC signal. Toward this end, for each trial, we convolved a duration-modulated boxcar with the HRF, renormalized the peak to unity, multiplied the regressor by the SCEPTIC signal on that trial, and then summed across trials to derive a single model-based regressor [see processing time versus intensity of activation in (*105*)].

Parameter estimates from each run were combined using a weighted fixed effects model in FEAT that propagated error variances from the individual runs. The contrasts from the second-level analyses were then analyzed at the group level using a mixed effects approach implemented in FSL FLAME. Specifically, we used the FLAME 1+2 approach with automatic outlier deweighting (*106*), which implements Bayesian mixed effects estimation of the group parameter estimates including full Markov chain Monte Carlo–based estimation for near-threshold voxels (*107*). To identify statistical parametric maps that best represented the average response, all group analyses included age and sex as covariates of no interest (especially given the developmental sample).

Although our analyses focus primarily on the DAN as the a priori network of interest, we nevertheless conducted whole-brain corrections to the voxelwise GLM statistics to examine the pattern of activity for the signals of interest. Specifically, to correct for family wise error at the whole-brain level, we applied the probabilistic threshold-free cluster enhancement methods (*108*), thresholding whole-brain maps at family-wise error-corrected *P* < 0.05 (e.g., Fig. 2B). This algorithm provides strict control over familywise error and boosts sensitivity to clusters of activated voxels.

#### Brain-behavior fMRI analyses using regression coefficients from model-based fMRI GLM analyses

To relate individual differences in entropy- and entropy change–related BOLD modulation to behavior on the clock task, we extracted subject-level parameter estimates for these GLM contrasts from each of the 47 DAN parcels defined by the Schaefer cortical parcellation [see table S2 and (*36*)]. These parameter estimates (also known as “betas”) served as individual difference measures of sensitivity to signals from SCEPTIC—particularly entropy and entropy change—across regions in the DAN.

We then entered DAN betas for SCEPTIC entropy change as a cross-level moderator of trial-level effects in multilevel models of behavior. Specifically, the dependent variable was trial-wise RT (choice) with behavioral variables as predictors. All models included the inverse-transformed trial number and previous reward as covariates. Models also included the influence of previous choice (RT_*t*−1_) on current choice (RT*_t_*) or RT autocorrelation. A weaker autocorrelation indicates larger exploratory RT swings, and variables that decrease autocorrelation are considered to increase exploration. Most models included DAN entropy change betas as cross-level moderators of the RT_*t*−1_ effect as a test of how sensitivity to updates in the number of good options modulated exploration on the task. Likewise, most multilevel models also included the trial-varying location of the best option, RT_Vmax_. The two-way interaction of RT_Vmax_ and DAN entropy change betas tests whether sensitivity to entropy change enhances or diminishes exploitation of the best option.

Because our behavioral observations had a clustered structure (i.e., trials nested within subjects), we used multilevel regression models, which were estimated using restricted maximum likelihood in the lme4 package (*109*) in R 4.2.0 (*110*). Estimated *P* values for predictors in the model were computed using Wald chi-square tests, and degrees of freedom were based on the Kenward-Roger approximation. For trial-level analyses, subject and run were treated as random and random intercepts were included for these factors. In addition, as noted in Results, we included random slopes of key terms such as RT_Vmax_ and RT_*t*−1_ to ensure the robustness of DAN modulation of exploitation and exploration (*111*).

#### Within-trial mixed-effects survival analyses of behavior with time-varying value estimates

To examine the sensitivity of choices to within-trial time-varying value, we performed survival analyses predicting the temporal occurrence of response. These mixed-effects Cox models (R coxme package) (*112*) estimated response hazard as a function of model-predicted expected value and their interaction with session-level DAN responses. This survival analysis does not assume that one precommits to a given RT; instead, modeling the within-trial response hazard function in real continuous time accounts for censoring and allows for a completely general baseline hazard function (*113*). The survival approach accounts for censoring of later within-trial time points by early responses. It allows for a completely general baseline hazard function that can vary randomly across participants. We thus avoid assumptions about the statistical distribution of RTs and account for trial-invariant influences such as urgency, processing speed constraints, or opportunity cost. We also modeled only the 1000- to 3500-ms interval, excluding early RTs that may be shorter than the deliberation and motor planning period and the end of the interval that one may avoid to not miss responding on a trial. We included learned value from the information-compressing model as a time-varying covariate, sampled every 100 ms. To account for between-person heterogeneity, person-specific intercept was included as a random effects; sensitivity analyses also included the random slope of the predictor of interest (value).

#### Analyses of within-trial peri-feedback BOLD responses using voxelwise deconvolution

Although betas from fMRI GLMs provide a useful window into how decision signals from SCEPTIC relate to behavior at the level of an entire session, the GLM approach makes a number of assumptions: (i) that one correctly specifies when in time a signal derived from a computational model modulates neural activity, (ii) that there is a linear relationship between the model signal and BOLD activity, and (iii) that a canonical HRF describes the BOLD activity corresponding to a given model-based signal. Furthermore, a conventional model-based fMRI GLM does not allow one to interrogate whether the representation of a given cognitive process varies in time over the course of a trial. For these reasons, we conducted additional analyses that could provide a detailed view of how DAN activity changes following feedback on each trial of the clock task. These analyses also attempted to overcome statistical and conceptual limitations of the GLM and to provide an index of within-trial neural activity that was independent of our computational model. That is, in these analyses, within-trial BOLD activity is the dependent variable and parameters from the SCEPTIC model are predictors.

We first applied a leading hemodynamic deconvolution algorithm to estimate neural activity from BOLD data (*114*). This algorithm has performed better than alternatives in simulated and real fMRI data, and it is reasonably robust to variations in the timing of neural events and the sampling frequency of the scan (*115*). We deconvolved the voxelwise BOLD activity for all subjects, averaged the deconvolved time series within each of the 47 DAN parcels (table S2), and retained these as a regions × time matrix for each run of fMRI data.

Then, to estimate neural activity for each trial in the experiment, we extracted the deconvolved signal surrounding feedback onset (−4 to +4 s), censoring time points that intersected the previous or next trials. Last, to ensure that discrete-time models of neural activity could be easily applied, we resampled deconvolved neural activity onto an evenly spaced grid aligned to the feedback onset using linear interpolation. The sampling frequency of the feedback-aligned deconvolved signals was matched to the TR of the fMRI scan (1 s for the original sample and 0.6 s for the replication sample). This interpolation was a form of resampling but did not upsample or downsample the data in the time domain.

For each subject, this yielded a 400 trial × 9 time point (−4 to 4 s for the main sample) × 47 region matrix. We then concatenated these matrices across participants for group analysis. Our primary analyses focused on the four parcels of the DAN visuomotor gradient (MT+, caudal PPC, rostral PPC, and premotor) rather than analyzing each region separately. Within each time × parcel combination, we regressed trialwise neural activity on key decision variables in a multilevel regression framework implemented in lme4 (*109*) in R, allowing for a random intercept of subject and random slopes for absolute reward prediction error [abs(PE)], entropy, entropy change, and *V*_max_. Multilevel models included the following predictors of activity: (negative) inverse-transformed trial number, RT, RT_*t*−1_, *V*_max_, side (left/right), entropy, entropy change, abs(PE), reward/omission, and the log-transformed Kullback-Leibler divergence between the last and three preceding RTs to account for stochastic choice histories. We note that all key effects hold when tested in simpler models with fewer simultaneous covariates. We also tested model variants that included a linear, rather than inverse-transformed, effect of trial but found that these fit worse, consistent with a disproportionate impact of earlier trials on neural activity in the DAN. Within this framework, the regression coefficients provide an estimate of when and in what region key signals such as entropy change are associated with feedback-related changes in neural activity.

Critically, however, given the temporal smoothness of BOLD data, the deconvolved signals remain highly autocorrelated, and we are cautious about overinterpreting the temporal precision of these analyses. Moreover, this temporal (and potentially spatial) association results in nonindependent statistical tests across the set of space × time models. To adjust for multiple comparisons in nonindependent models, we applied the Benjamini-Yekutieli correction across terms of interest in these models to maintain an FDR of 0.05 (*116*).

Another advantage of this analytic approach is that alternative models of representation and behavior can be compared in terms of their alignment to neural activity in fMRI. More specifically, each multilevel model across the space × time set of models yields global fit measures such as the AIC, which can be used to compare the relative fit of cognitive signals (e.g., entropy change) to event-aligned BOLD data. Here, we used a global model selection approach (*117*) based on the AIC to compare the fit of information-compressing, traditional RL, and WM accounts of the clock task to activity in the DAN (Fig. 3).

### MEG analyses

#### Multilevel analyses of time-frequency domain MEG data

The goal of these analyses was to estimate how reinforcement modulated oscillatory power at each within-trial time point and each frequency. To estimate this effect accurately and robustly across sensors and individuals, we used high-performance parallel computing to fit one multilevel regression model for each point in this time-frequency space, combining data from all trials, individuals, and sensors. Predictors included the SCEPTIC model–derived entropy change signal and behavioral confounds: current and previous RTs, reward/omission, inverse-transformed trial, and, in sensitivity analyses, the KL distance between the last and three preceding RTs to account for stochastic choice histories. Subject and sensor were treated as crossed random effects, with sensor-specific random intercepts and random slopes of the behavioral variable of interest and subject-specific random intercepts and, where indicated, random slopes of the variable of interest. Because our contrasts were between trials, the intercept accounted for marginal oscillatory power at a given time-frequency point, and correction for baseline was not necessary. Models were estimated using restricted maximum likelihood in the lme4 package (*109*) in R 4.2.0 (*110*). Estimated *P* values for predictors in the model were computed using Wald chi-square tests, and degrees of freedom were based on the Kenward-Roger approximation. To examine the anatomical distribution of effects, after obtaining estimates for each subject and sensor within this overall model, we projected them into the sensor space (Fig. 5C) and source space (Fig. 5D) as follows.

Source location was performed using the linearly constrained minimum variance (LCMV) Beamformer procedure (*118*). We used Freesurfer’s “fsaverage” template source space and sensor-to-template registration provided by the MNE software (*119*). The forward model was calculated using the single-layer boundary element, for a total of 20,484 potential source locations placed with 5-mm spacing on the fsaverage surface. A spatial filter was then constructed using a unit-gain LCMV Beamformer (*118*), with covariances estimated using the 1-s window from the peristimulus interval and 1-s window after the feedback presentation, across all trials and all subjects. We applied the filter to project sensor-level group statistics to the source space. Source estimates were thresholded from 20th to 95th percentiles.

Our analyses of the relationship between subject-level oscillatory responses and behavioral exploration/exploitation used multilevel survival models identical to those described above (“Within-trial mixed-effects survival analyses of behavior with time-varying value estimates” section).
