# Supplementary material for: Reward-based option competition in human dorsal stream and transition from stochastic exploration to exploitation in continuous space
Source: Sci Adv. 2024 Feb 23;10(8):eadj2219. doi: 10.1126/sciadv.adj2219 (PMC10889364; doi:10.1126/sciadv.adj2219)
Supplement: Supplementary file 1 — Figs. S1 to S7 Tables S1 to S5 [file sciadv.adj2219_sm.pdf]

Science Advances
AAAS

Supplementary Materials for

Reward-based option competition in human dorsal stream and transition from stochastic exploration to exploitation in continuous space

Michael N. Hallquist *et al.*

Corresponding author: Alexandre Y. Dombrovski, dombrovskia@gmail.com

Sci. Adv. **10**, eadj2219 (2024)
DOI: 10.1126/sciadv.adj2219

This PDF file includes:

Figs. S1 to S7
Tables S1 to S5

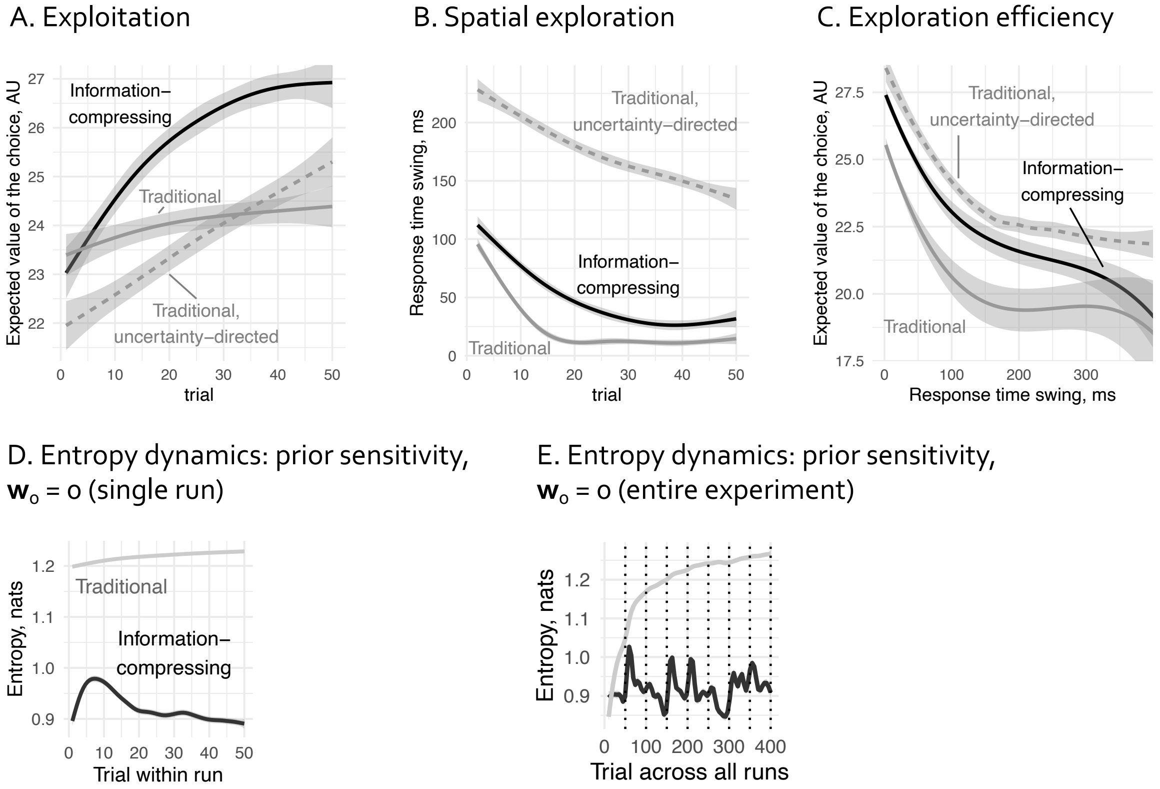

Figure S1. Information-compressing RL model: qualitative behavioral predictions and prior sensitivity checks.

(A) Exploitation: expected reward (value) by trial. Information-compressing (black) and traditional RL (grey, solid) algorithms are compared to more sophisticated but costly uncertainty-directed exploration (grey, dashed). Information-compressing RL model (black) engages in successful exploitation of the continuous space reaching an early, high asymptote. Traditional RL (grey, solid) also asymptotes, but at a lower value. Returns for traditional RL with uncertainty-directed exploration (grey, dashed) continue to improve, but the overall cost of exploration is very high. Data from a computational study described in detail in Hallquist & Dombrovski, Cognition, 2019 examining model behavior in 40 simulated environments across different parameter sets.

(B) Exploration of the continuous space measured by the absolute distance between consecutive choices (response time swings on the clock task. Traditional RL (grey, solid) decreases its exploration rate early due to long-term value persistence, while information-compressing RL (black) displays more persistent exploration. Uncertainty-directed traditional RL (grey, dashed) displays the highest exploration rate.

(C) Exploration efficiency, illustrating expected reward (ordinate) as a function of the absolute distance between consecutive choices (abscissa). Information-compressing RL (black) explores nearly as efficiently as uncertainty-directed RL (grey, dashed), while traditional RL (grey, solid) explores less efficiently.

(D) Entropy dynamics of the information-compressing vs. traditional RL model across an average run. When prior basis element weights are set to 0 instead of random uniform (cf Figure 1G), the dynamics remain qualitatively unchanged.

(E) The same as D, across the entire experiment (cf Figure 1H).

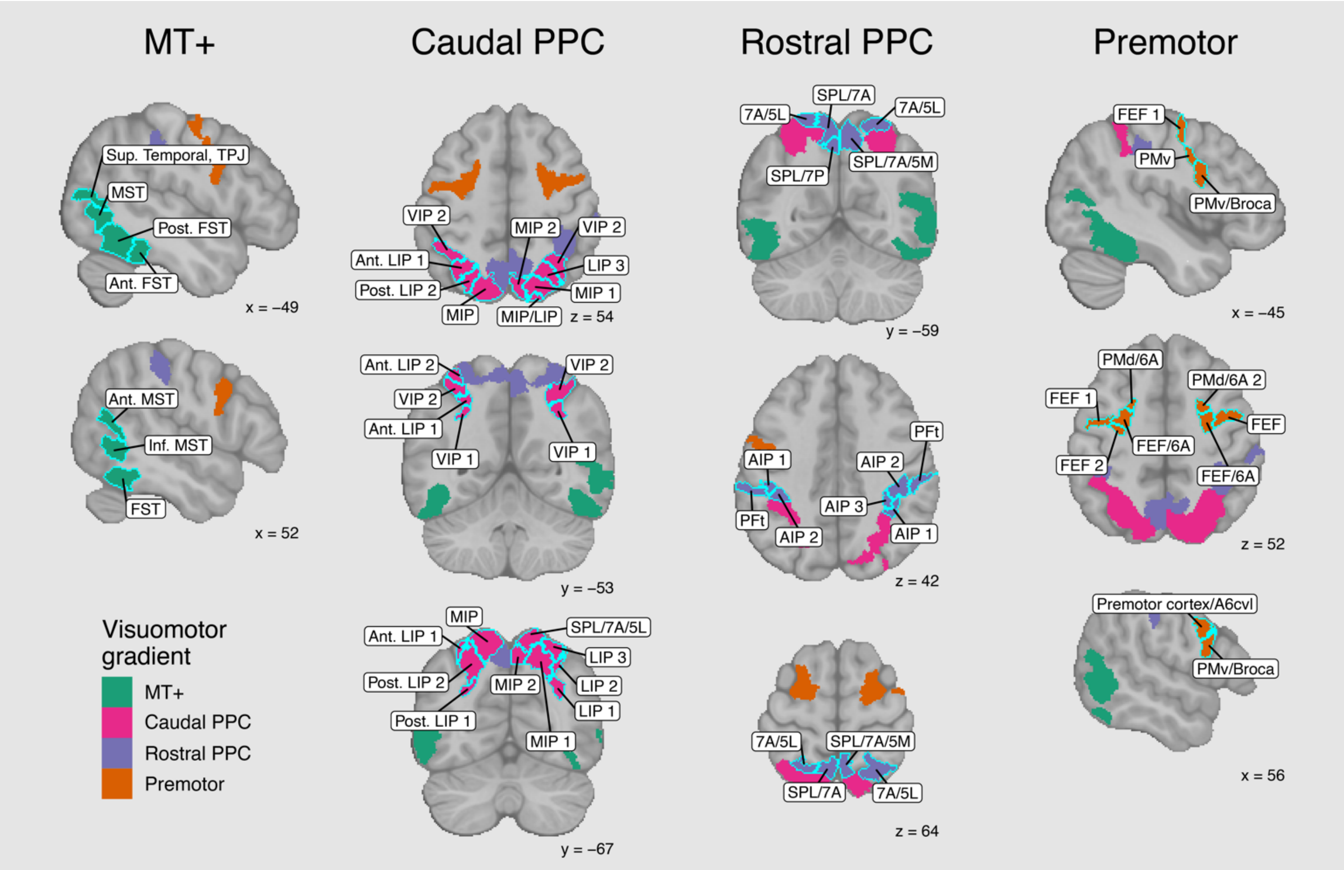

Figure S2. Connectivity-based parcellation of the dorsal stream: individual parcels labeled.

Columns are arranged from late visual to premotor regions. Descriptive labels are provided for comparison to non-human primate studies. A6cvl = caudal ventrolateral area 6; Brodmann AIP = anterior intraparietal; FEF = frontal eye field; FST = fundus of the superior temporal area; LIP = lateral intraparietal; MIP = medial intraparietal; MT+ = motion-sensitive middle temporal visual area 5; MST = medial superior temporal area; PFt = rostral intraparietal lobule area PFt; PMd = dorsal premotor; PMv = ventral premotor; SPL = superior parietal lobule.

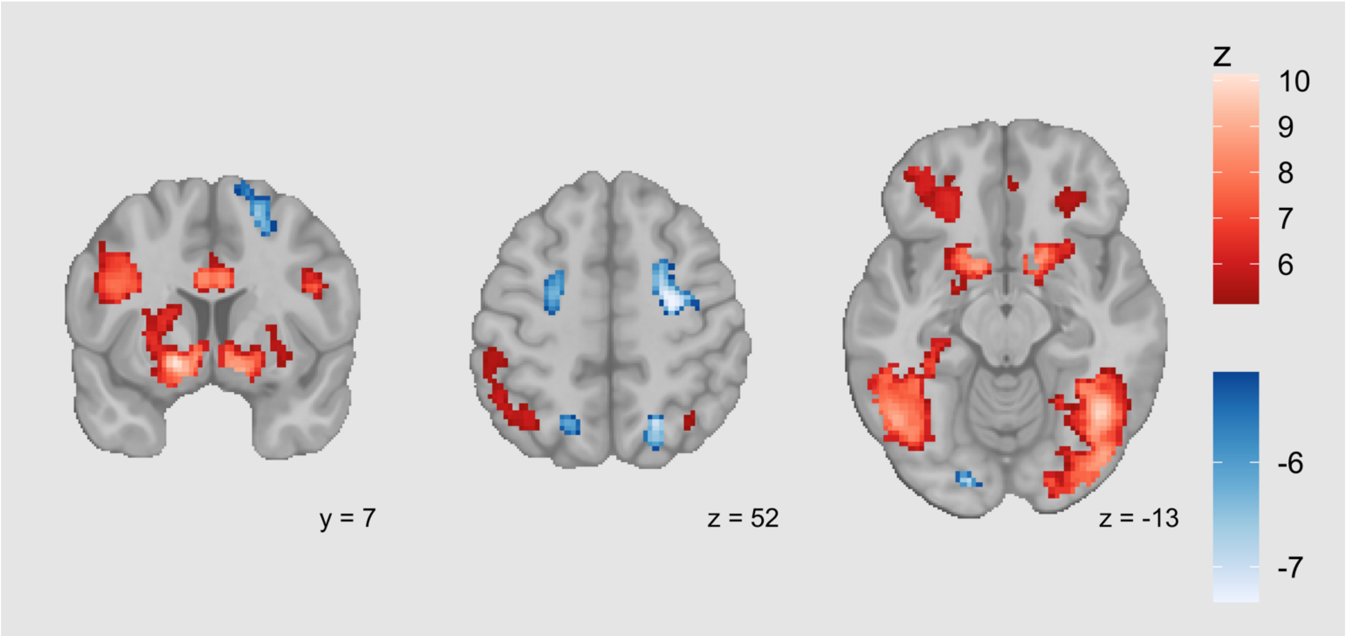

Figure S3. Responses to reward (red) vs. reward omission (blue), whole-brain analysis.

As expected, rewards elicit responses in the striatum (left) and orbitofrontal cortex (right), but also in MT+ (right). Reward omissions elicit responses in the frontoparietal nodes of the dorsal attention network (center).

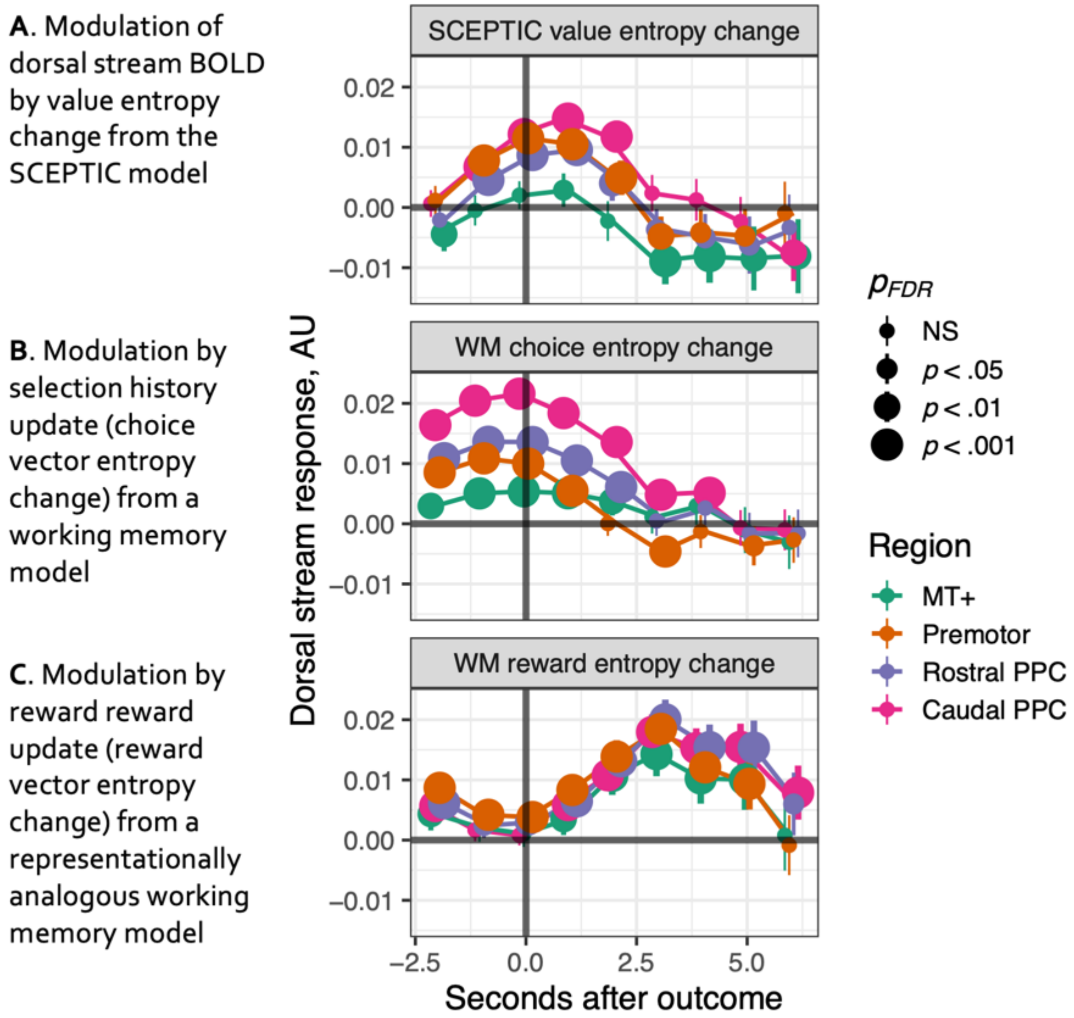

Figure S4. Information dynamics of the value function, DAN BOLD signal.

(A) Modulation by SCEPTIC-predicted value entropy change persisted after controlling for the selection history update **(B)** and reward history update **(C)** from a representationally analogous working memory model. While BOLD modulation by selection history update occurred peri-response (B), responses to value entropy changed peaked around 1s (A) while responses to reward history update peaked around 3s. Error bars denote the standard error of the estimate from the multilevel model.

A. Responses to entropy change, model without covariates

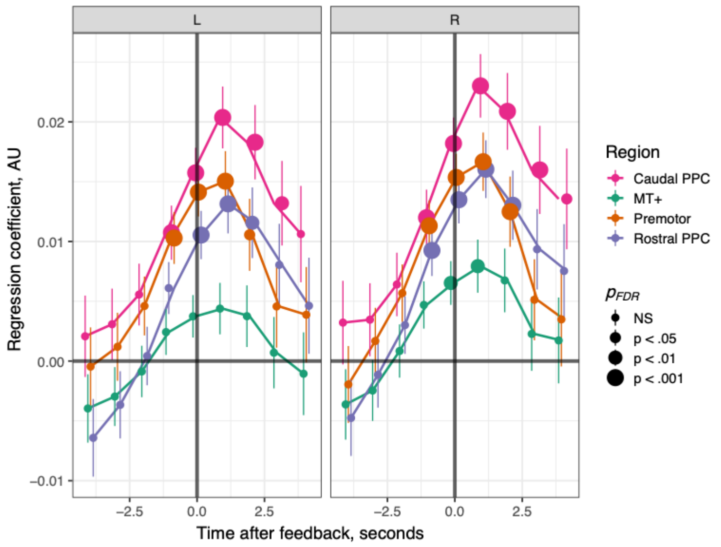

B. Effect of preceding RT swings: modulation by summed Kullback-Leibler divergence (KLD) of response times for trials t-4, t-3, and t-2 versus the response times of the preceding three trials

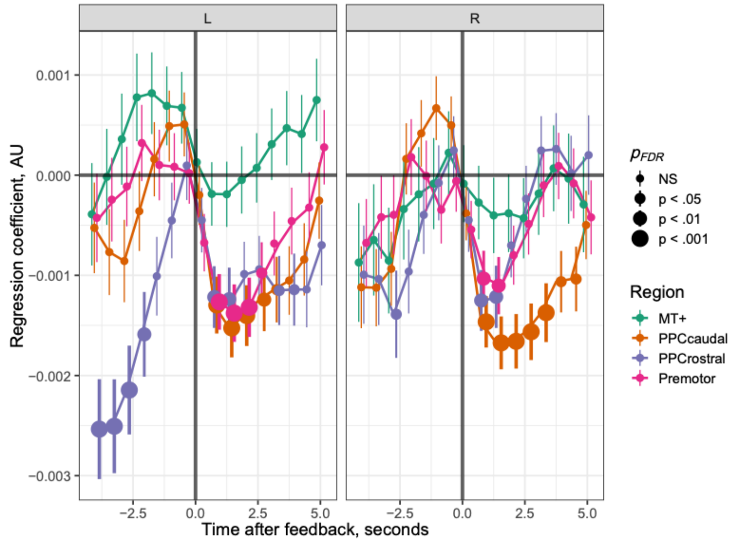

Figure S5. DAN deconvolved BOLD response to entropy increases: sensitivity analyses

(A) Model predicting DAN deconvolved BOLD signal with entropy change without controlling for behavioral confounds (current and previous RT, reward/omission, prediction error, $V_{max,}$ entropy).

(B) Selection history may impact value entropy and confound its neural correlates. To rule out this possibility, we computed the summed Kullback-Leibler divergence (KLD) of response times for trials t-4, t-3, and t-2 from the local distribution of response times of the preceding three trials. Higher values of this measure reflect a history of larger RT swings.

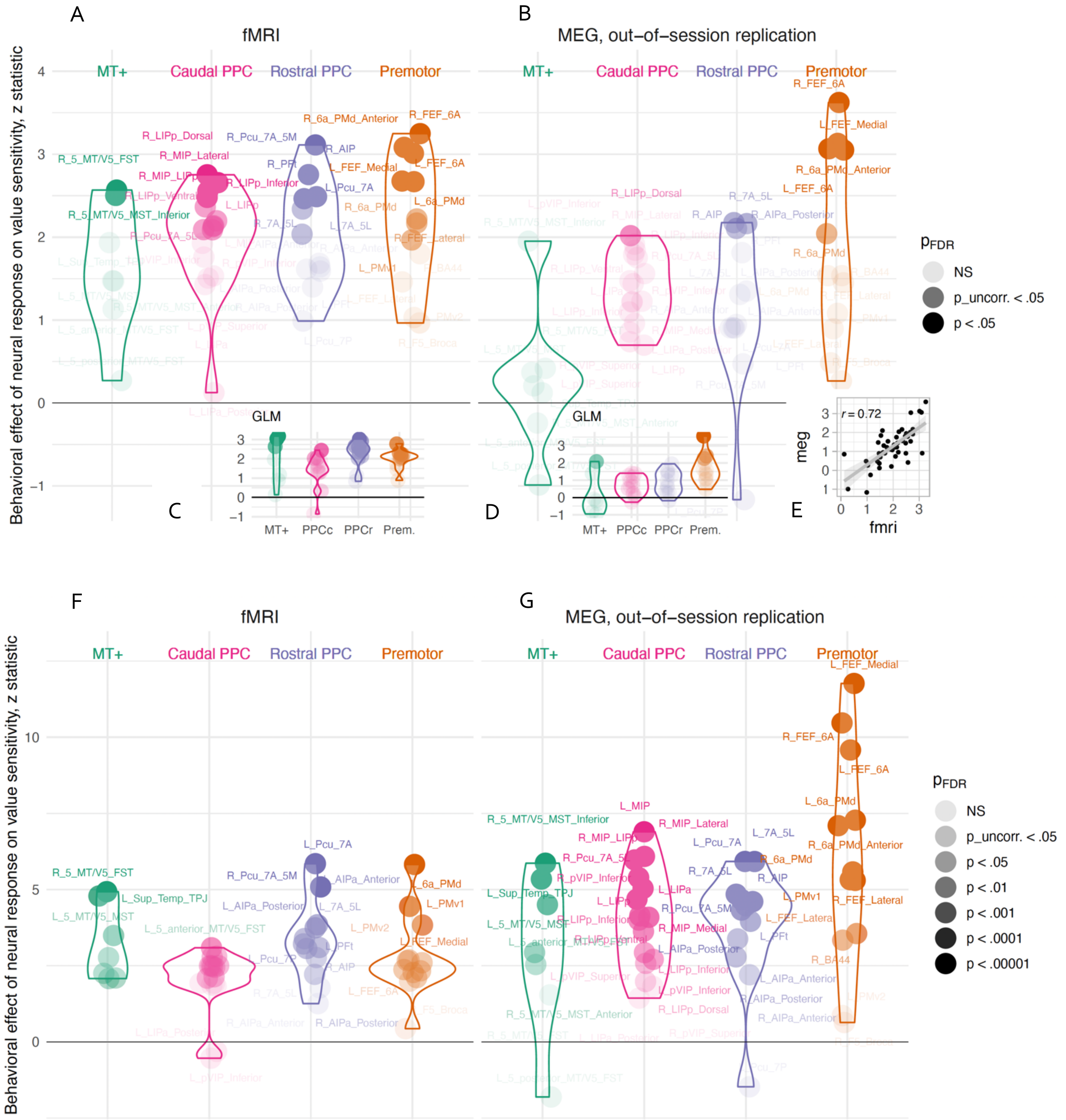
A
fMRI
B
MEG, out−of−session replication
MT+
Caudal PPC
Rostral PPC
Premotor
Behavioral effect of neural response on value sensitivity, z statistic
GLM
C
D
E
MT+
PPCc
PPCr
Prem.
r = 0.72
meg
fmri
pFDR
NS
p_uncorr. < .05
p < .05
F
G
p < .01
p < .001
p < .0001
p < .00001

Figure S6. BOLD encoding of value information dynamics and behavioral exploitation, sensitivity analyses

Multi-level survival analyses examining how the individual's neural response moderates their behavioral sensitivity to within-trial time-varying value. A-D: Models include individual random slopes of value to account for between-persons heterogeneity. F-G: Same model as in Figure 4 but censoring the first 1s and last 0.5s of the interval.

(A) Original fMRI session.

(B) Replication, MEG session. Greater modulation of individual DAN BOLD response by value entropy change predicted more exploitative choices.

(C), (D) Same, GLM analysis.

(E) The anatomical pattern of brain-behavior associations was preserved across the original fMRI and replication sessions. Each dot represents a single DAN parcel as labeled in panels A, B.

(F) Original fMRI session, censoring the first 1s and last 0.5s of the interval.

(G) Replication, MEG session, censoring the first 1s and last 0.5s of the interval.

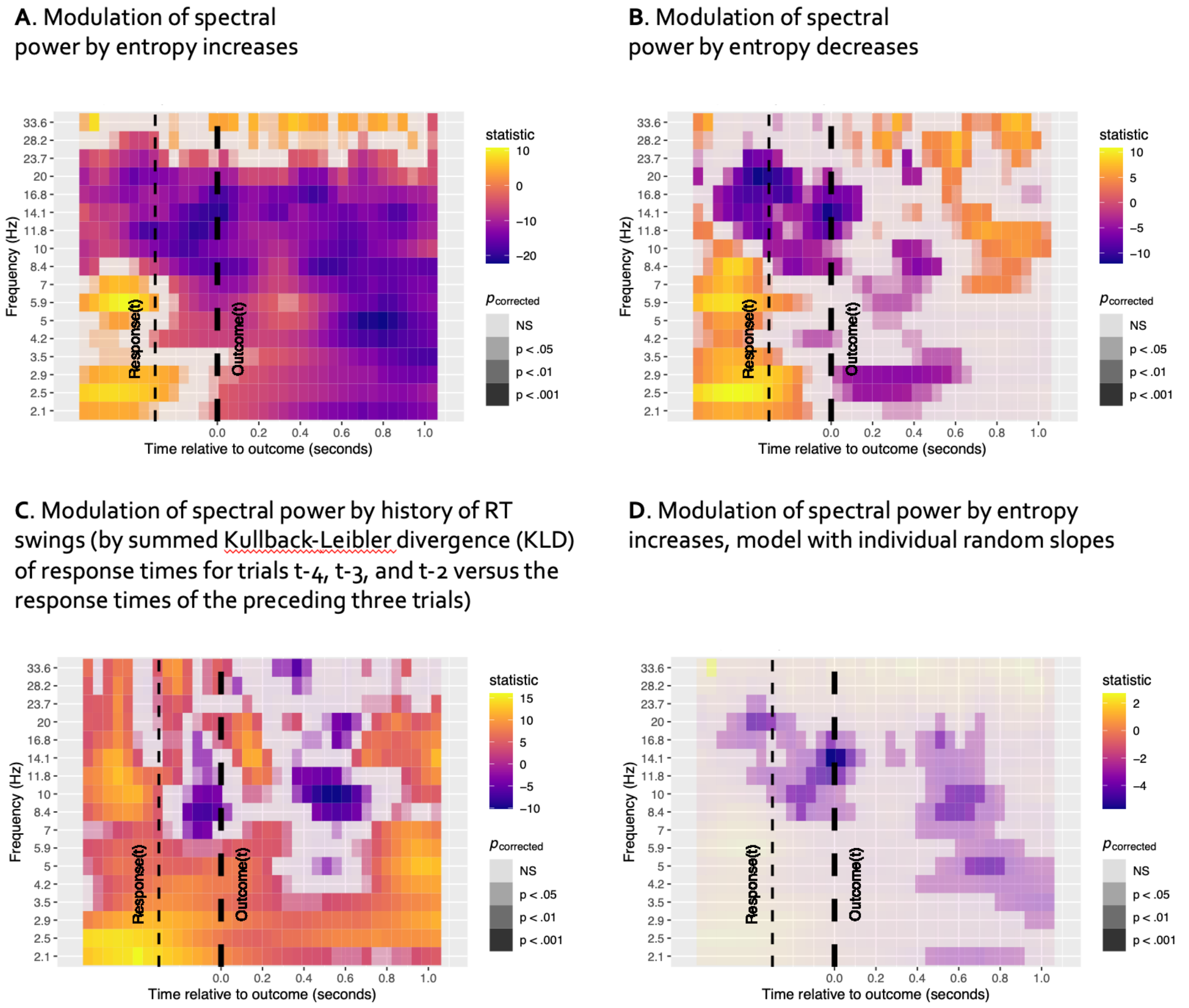

Figure S7. Suppression of low beta and high alpha oscillatory power in response to entropy increases, sensitivity analyses.

(A) Oscillatory response to value entropy increases only, with any entropy decrease coded as 0. Cool colors represent de-synchronization to increases reflecting a rising number of potentially valuable options, most prominent between 7-17 Hz (β_1/α band) at 400-750 ms. The result is nearly identical to the model reported in Figure 5A, which used a full directional entropy change predictor.

(B) Oscillatory response to value entropy decreases only, with any entropy increase coded as 0. The β_1/α response at 400-750 ms is lacking.

(C) Oscillatory response to RT swings. The The β_1/α response at 400-750 ms is similar albeit weaker than that elicited by entropy change, suggesting that β_1/α responses encode both reinforcement and selection history.

(D) Oscillatory response to value entropy change, multi-level model including individual random slopes of entropy change to account for between-subjects heterogeneity.

Table S1. Behavior during the fMRI and MEG sessions

	fMRI session				MEG session				Contrast, MEG - fMRI	
Predictors	*Estimates*	*std. Error*	*Statistic*	*p*	*Estimates*	*std. Error*	*Statistic*	*p*	*Statistic*	*p*
Intercept	-0.70	0.03	-23.79	**<0.001**	-0.71	0.03	-26.85	**<0.001**	0.93	0.350
RT_lag	0.46	0.01	64.68	**<0.001**	0.45	0.01	74.00	**<0.001**	-3.21	**0.001**
Omission_lag	0.44	0.02	22.50	**<0.001**	0.38	0.02	22.89	**<0.001**	-3.39	**0.001**
RT_Vmax	0.13	0.01	21.24	**<0.001**	0.16	0.01	29.79	**<0.001**	2.85	**0.004**
Trial	0.04	0.01	4.84	**<0.001**	0.08	0.01	11.04	**<0.001**	4.00	**<0.001**
RT_lag * Omission_lag	-0.34	0.01	-36.93	**<0.001**	-0.29	0.01	-37.79	**<0.001**	5.35	**<0.001**
RT_Vmax * Trial	0.05	0.01	6.35	**<0.001**	0.06	0.01	8.46	**<0.001**	0.71	0.481
Observations	29239				34940				64179	
Marginal R^2 / Conditional R^2	0.225 / 0.351				0.296 / 0.407				0.346 / 0.398	

Note. Statistics from multi-level models predicting participants' choices (response times, RT) with the preceding RT (RT_lag, with a lower coefficient reflecting greater RT swings), previous reward vs. omission (Omission_lag), highest-valued RT predicted by the SCEPTIC model (RT_Vmax, reflecting exploitation), trial (negative inverse-transformed and scaled based on Hallquist & Dombrovski, Cognition, 2019), RT_lag * Omission_lag interaction (capturing win-stay/lose-shift behavior) and RT_Vmax * Trial interaction (capturing greater exploitation late in learning). Note that (i) effects of exploitation explain the decrease in exploration seen in raw data (Figure 1D) and (ii) generally stronger statistics during the MEG session mostly reflect a higher number of trials (540 vs. 400 in fMRI). Overall, while participants performed slightly better (lower exploration, higher exploitation, weaker win-stay/lose-shift tendency) in the more comfortable MEG lab compared to the MRI scanner, these differences reached statistical significance mostly due to the increased sample size in the pooled analysis whereas behavior was qualitatively similar.

Table S2. Schaefer 2018 parcellation of dorsal attention network (DAN) regions

Schaefer node	Region label	Visuomotor grouping	Hemisphere	MNI x	MNI y	MNI z
69	Anterior FST	MT+	L	-44	-42	-21
70	Posterior FST	MT+	L	-48	-55	-15
71	MST	MT+	L	-54	-62	-1
72	Superior Temporal, TPJ	MT+	L	-46	-68	14
73	Posterior LIP 1	Caudal PPC	L	-27	-70	29
74	PFt	Rostral PPC	L	-53	-31	43
75	Posterior LIP 2	Caudal PPC	L	-24	-64	45
76	AIP 1	Rostral PPC	L	-43	-30	39
77	VIP 1	Caudal PPC	L	-33	-46	39
78	Anterior LIP 1	Caudal PPC	L	-28	-58	48
79	AIP 2	Rostral PPC	L	-36	-37	44
80	VIP 2	Caudal PPC	L	-35	-50	54
81	MIP	Caudal PPC	L	-15	-69	56
82	Anterior LIP 2	Caudal PPC	L	-27	-58	60
83	SPL/7A	Rostral PPC	L	-7	-57	61
84	7A/5L	Rostral PPC	L	-20	-54	65
86	FEF 1	Premotor	L	-40	-4	51
87	FEF/6A	Premotor	L	-26	-2	52
88	FEF 2	Premotor	L	-30	-9	49
89	PMd/6A	Premotor	L	-21	3	62
90	PMv/Broca	Premotor	L	-48	6	25
91	PMv	Premotor	L	-49	1	37
145	SPL/7P	Rostral PPC	L	-4	-62	51
245	Premotor cortex/A6cvl	Premotor	R	59	6	31
271	FST	MT+	R	48	-51	-17

272	Inferior MST	MT+	R	58	-56	-1
273	Anterior MST	MT+	R	52	-57	12
275	LIP 1	Caudal PPC	R	31	-67	35
276	PFt	Rostral PPC	R	55	-25	44
277	MIP/LIP	Caudal PPC	R	17	-76	48
278	AIP 1	Rostral PPC	R	38	-39	38
279	AIP 2	Rostral PPC	R	43	-29	40
280	LIP 2	Caudal PPC	R	28	-61	49
281	VIP 1	Caudal PPC	R	32	-46	41
282	MIP 1	Caudal PPC	R	20	-68	53
283	AIP 3	Rostral PPC	R	38	-38	49
284	VIP 2	Caudal PPC	R	33	-52	53
285	MIP 2	Caudal PPC	R	7	-70	52
286	LIP 3	Caudal PPC	R	26	-58	59
287	SPL/7A/5M	Rostral PPC	R	7	-53	57
288	SPL/7A/5L	Caudal PPC	R	13	-63	63
289	7A/5L	Rostral PPC	R	25	-51	66
290	FEF	Premotor	R	39	-3	51
291	FEF/6A	Premotor	R	27	-4	49
292	PMd/6A 1	Premotor	R	24	-4	61
293	PMv/Broca	Premotor	R	48	8	26
355	PMd/6A 2	Premotor	R	24	8	57

Note. These regions were extracted from the 400-node cortical parcellation of Schaefer and colleagues (2018). The nodes included in the DAN differ slightly depending on whether one considers a 7-network or 17-network parcellation. Moreover, we compared DAN nodes in the 200-node variant of the parcellation to ensure robustness of the network definition. The 47 nodes included here are the union of regions contained in the DAN, as defined by the 200- and 400-node solutions, as well as the 7- and 17-network assignments. The Schaefer node numbers reflect the node numbering scheme from the 400-node, 7-network solution. A6cvl = caudal ventrolateral area 6; Brodmann AIP = anterior intraparietal; FEF = frontal eye field; FST = fundus of the superior temporal area; LIP = lateral intraparietal; MIP = medial intraparietal; MT+ = motion-sensitive middle temporal visual area 5; MST = medial superior temporal area; PFt = rostral intraparietal lobule area PFt; PMd = dorsal premotor; PMv = ventral premotor; SPL = superior parietal lobule.

Table S3. Clusters of voxels significantly modulated by entropy in whole-brain analyses.

Label	Network	Hemi-sphere	x	y	z	Voxels	*M z*
Area 9 Middle	Default	L	-7	56	8	159	-5.83
Area s32	Default	R	5	40	-10	136	-5.64
Area 10r	Default	L	-5	54	-10	104	-5.92
Area a24	Default	L	-6	34	-9	92	-5.78
Superior 6-8 Transitional Area	Default	R	23	23	51	60	5.90
PreCuneus Visual Area	Default	R	6	-48	44	57	6.33
Lateral Area 7P	Dorsal Attention	L	-15	-69	56	330	8.28
Medial IntraParietal Area	Dorsal Attention	R	20	-68	53	248	8.98
PreCuneus Visual Area	Dorsal Attention	R	7	-53	57	191	8.15
Anterior IntraParietal Area	Dorsal Attention	R	38	-38	49	167	7.45
Area Lateral IntraParietal ventral	Dorsal Attention	R	26	-58	59	151	8.26
Area Lateral IntraParietal ventral	Dorsal Attention	R	33	-52	53	128	7.47
Medial Area 7P	Dorsal Attention	R	7	-70	52	122	9.03
Lateral Area 7A	Dorsal Attention	R	25	-51	66	121	7.69
Ventral IntraParietal Complex	Dorsal Attention	L	-27	-58	60	108	7.06
Medial Area 7A	Dorsal Attention	R	13	-63	63	103	9.57
Medial IntraParietal Area	Dorsal Attention	L	-24	-64	45	96	6.91
Medial Area 7A	Dorsal Attention	L	-7	-57	61	96	6.98
Area 6 anterior	Dorsal Attention	R	27	-4	49	89	7.40
Anterior IntraParietal Area	Dorsal Attention	R	38	-39	38	77	7.77
Dorsal Transitional Visual Area	Dorsal Attention	R	17	-76	48	71	7.92
Area Lateral IntraParietal ventral	Dorsal Attention	L	-35	-50	54	68	6.23
Area PGp	Dorsal Attention	R	43	-76	31	68	6.54
Area 6 anterior	Dorsal Attention	L	-26	-2	52	55	5.57

Label	Network	Hemi-sphere	x	y	z	Voxels	*M z*
Cerebellum	Frontoparietal Control	L	-31	-65	-38	345	5.93
Area IntraParietal 2	Frontoparietal Control	R	51	-36	50	134	6.65
Medial Area 7P	Frontoparietal Control	L	-4	-62	51	133	7.14
Inferior 6-8 Transitional Area	Frontoparietal Control	R	33	14	54	127	6.44
Area 6m anterior	Frontoparietal Control	R	24	8	57	114	7.11
Area PFm Complex	Frontoparietal Control	R	43	-47	47	80	7.54
Area 7m	Frontoparietal Control	R	6	-62	44	66	6.49
Primary Motor Cortex	Somatomotor	L	-34	-19	62	95	-5.39
Primary Sensory Cortex	Somatomotor	L	-32	-30	60	53	-5.37
Cerebellum	Ventral Attention	L	-25	-56	-37	174	5.80
IntraParietal Sulcus Area 1	Visual	L	-21	-77	43	87	6.67

Note. Entropy from the SCEPTIC model was aligned to onset of the decision (clock) phase. Clusters of whole-brain-significant voxels were identified based on a familywise error rate of .05 using the probabilistic threshold-free cluster enhancement (pTFCE) approach. These clusters were then divided into functional regions by calculating the overlap between each data-derived cluster and a whole-brain parcellation adapted from Schaefer and colleagues (2018), detailed in Methods and can be found in the Dryad dataset linked with this article: 10.5061/dryad.hmgqnk9qc. Regions in the parcellation that overlapped with the data-derived clusters by at least 50 voxels are displayed in the table. Spatial coordinates are reported in MNI152 stereotaxic space. Regions are labeled using Glasser and colleagues' 2016 BALSA parcellation. Voxels were 2.3mm^3 in size.

Table S4. Clusters of voxels significantly modulated by entropy change in whole-brain analyses.

Label	Network	Hemi-sphere	x	y	z	n Voxels	Mean z
Superior Frontal Language Area	Default	R	12	18	62	221	8.57
Parieto-Occipital Sulcus Area 1	Default	R	12	-55	15	284	8.75
Parieto-Occipital Sulcus Area 2	Default	R	16	-63	28	106	9.48
IntraParietal Sulcus Area 1	Dorsal Attention	L	-27	-70	29	88	9.15
Area PFt	Dorsal Attention	L	-53	-31	43	131	8.85
Medial IntraParietal Area	Dorsal Attention	L	-24	-64	45	194	9.35
Anterior IntraParietal Area	Dorsal Attention	L	-43	-30	39	65	9.32
Area Lateral IntraParietal dorsal	Dorsal Attention	L	-33	-46	39	163	8.58
Area Lateral IntraParietal ventral	Dorsal Attention	L	-28	-58	48	175	8.93
Anterior IntraParietal Area	Dorsal Attention	L	-36	-37	44	138	9.20
Area Lateral IntraParietal ventral	Dorsal Attention	L	-35	-50	54	163	8.31
Lateral Area 7P	Dorsal Attention	L	-15	-69	56	405	9.57
Area 6 anterior	Dorsal Attention	L	-26	-2	52	99	10.30
Area 6 anterior	Dorsal Attention	L	-30	-9	49	57	9.28
Area 6 anterior	Dorsal Attention	L	-21	3	62	337	10.13
Rostral Area 6	Dorsal Attention	L	-48	6	25	237	8.88
Premotor Eye Fields	Dorsal Attention	L	-49	1	37	143	8.82
Area IntraParietal 1	Dorsal Attention	R	31	-67	35	121	8.84
Area PFt	Dorsal Attention	R	55	-25	44	163	8.51
Dorsal Transitional Visual Area	Dorsal Attention	R	17	-76	48	158	9.00
Anterior IntraParietal Area	Dorsal Attention	R	38	-39	38	80	8.45
Anterior IntraParietal Area	Dorsal Attention	R	43	-29	40	131	8.30
Area Lateral IntraParietal ventral	Dorsal Attention	R	28	-61	49	65	8.56
Medial IntraParietal Area	Dorsal Attention	R	20	-68	53	283	8.99

Label	Network	Hemi-sphere	x	y	z	n Voxels	Mean z
Anterior IntraParietal Area	Dorsal Attention	R	38	-38	49	286	8.19
Area Lateral IntraParietal ventral	Dorsal Attention	R	33	-52	53	197	8.03
Area Lateral IntraParietal ventral	Dorsal Attention	R	26	-58	59	171	8.24
PreCuneus Visual Area	Dorsal Attention	R	7	-53	57	242	8.34
Frontal Eye Fields	Dorsal Attention	R	39	-3	51	204	9.33
Area 6 anterior	Dorsal Attention	R	27	-4	49	92	9.77
Area 6 anterior	Dorsal Attention	R	24	-4	61	157	9.36
Rostral Area 6	Dorsal Attention	R	48	8	26	244	8.64
Anterior IntraParietal Area	Frontoparietal Control	L	-45	-43	47	131	9.00
Anterior Agranular Insula Complex	Frontoparietal Control	L	-34	17	-8	121	9.48
Area 33 prime	Frontoparietal Control	L	-3	4	29	90	8.48
Area IntraParietal 2	Frontoparietal Control	R	51	-36	50	225	7.98
Area PFm Complex	Frontoparietal Control	R	43	-47	47	91	8.02
Anterior Ventral Insular Area	Frontoparietal Control	R	34	22	-6	155	9.88
Area IFJa	Frontoparietal Control	R	46	18	25	157	9.26
Area 8C	Frontoparietal Control	R	37	11	32	120	8.70
Area 55b	Frontoparietal Control	R	40	7	50	126	9.14
Inferior 6-8 Transitional Area	Frontoparietal Control	R	33	14	54	286	9.01
Area 6m anterior	Frontoparietal Control	R	24	8	57	151	10.19
Area posterior 24	Frontoparietal Control	R	8	34	25	267	9.58
Area 8BM	Frontoparietal Control	R	5	26	47	222	9.82
Area OP2-3/VS	Somatomotor	L	-36	-26	19	135	7.81
Area 2	Somatomotor	L	-53	-20	39	129	8.44
Primary Sensory Cortex	Somatomotor	L	-47	-19	53	159	7.95
Area 1	Somatomotor	L	-46	-30	56	250	8.80
Area 3a	Somatomotor	L	-37	-26	49	45	8.28

Label	Network	Hemi-sphere	x	y	z	n Voxels	Mean z
Dorsal Area 24d	Somatomotor	L	-4	-9	57	174	8.71
Primary Motor Cortex	Somatomotor	L	-34	-19	62	288	8.01
Area 2	Somatomotor	L	-30	-45	60	244	7.88
Primary Sensory Cortex	Somatomotor	L	-32	-30	60	152	8.01
Area 6 anterior	Somatomotor	L	-25	-12	61	224	9.48
Area 6mp	Somatomotor	L	-15	-13	70	199	7.87
Area PFt	Somatomotor	R	60	-15	31	215	8.06
Area 2	Somatomotor	R	53	-17	40	112	8.30
Area 23c	Somatomotor	R	11	-16	41	118	7.31
Area 6mp	Somatomotor	R	27	-12	62	159	8.36
Anterior Ventral Insular Area	Ventral Attention	L	-32	26	0	138	9.73
Frontal Opercular Area 4	Ventral Attention	L	-32	20	9	159	9.08
Frontal Opercular Area 4	Ventral Attention	L	-43	14	3	128	8.44
Anterior 24 prime	Ventral Attention	L	-6	21	31	217	8.70
Ventral Area 24d	Ventral Attention	L	-6	8	47	177	9.78
Supplementary And Cingulate Eye	Ventral Attention	L	-8	-5	68	114	8.70
Area TemporoParietoOccipital Junction 1	Ventral Attention	R	50	-41	14	98	7.71
Superior Temporal Visual Area	Ventral Attention	R	61	-40	22	154	7.62
Area PF Complex	Ventral Attention	R	62	-28	39	378	7.88
Middle Insular Area	Ventral Attention	R	40	9	-1	168	8.38
Anterior Ventral Insular Area	Ventral Attention	R	36	23	6	265	9.79
Rostral Area 6	Ventral Attention	R	53	13	12	228	8.20
Anterior 24 prime	Ventral Attention	R	7	17	35	291	9.14
Area 23c	Ventral Attention	R	11	-32	42	187	7.66
Supplementary And Cingulate Eye	Ventral Attention	R	6	9	54	134	9.83
Area 23c	Ventral Attention	R	10	-43	53	116	8.52

Label	Network	Hemi-sphere	x	y	z	n Voxels	Mean z
Supplementary And Cingulate Eye	Ventral Attention	R	6	-3	65	219	8.92
Area 6m anterior	Ventral Attention	R	16	4	67	137	9.95
Second Visual Area	Visual	L	-20	-65	7	158	6.96
Second Visual Area	Visual	R	16	-65	19	223	8.62

Note. Entropy change from the SCEPTIC model was aligned to onset of the feedback, representing change in entropy due to the reinforcement. Clusters of whole-brain-significant voxels were identified based on a familywise error rate of .05 using the probabilistic threshold-free cluster enhancement (pTFCE) approach. These clusters were then divided into functional regions by calculating the overlap between each data-derived cluster and a whole-brain parcellation adapted from Schaefer and colleagues (2018), detailed in Methods and can be found in the Dryad dataset linked with this article: 10.5061/dryad.hmgqnk9qc. Regions in the parcellation that overlapped with the data-derived clusters by at least 75% are displayed in the table. Spatial coordinates are reported in MNI152 stereotaxic space. Regions are labeled using Glasser and colleagues' 2016 BALSA parcellation. Voxels were $2.3mm^3$ in size.

Table S5. Effects of reinforcement history on choice beyond the timeframe of the working memory buffer.

	fMRI session		MEG session	
Predictors	*Statistic*	*p*	*Statistic*	*p*
Intercept	-24.43	**<0.001**	-32.02	**<0.001**
Trial	-1.17	0.242	-1.60	0.109
RT_lag1	54.11	**<0.001**	64.10	**<0.001**
RT_lag2	13.98	**<0.001**	18.15	**<0.001**
RT_lag3	7.75	**<0.001**	6.19	**<0.001**
RT_lag4	2.65	**0.008**	4.94	**<0.001**
RT_lag5	1.73	0.084	3.92	**<0.001**
Omission_lag1	20.47	**<0.001**	21.56	**<0.001**
Omission_lag2	13.01	**<0.001**	12.96	**<0.001**
Omission_lag3	6.50	**<0.001**	5.55	**<0.001**
Omission_lag4	3.09	**0.002**	2.67	**0.008**
Omission_lag5	-0.36	0.720	-1.30	0.194
RT_lag1 * Omission_lag1	-34.49	**<0.001**	-36.14	**<0.001**
RT_lag2 * Omission_lag2	-16.00	**<0.001**	-16.66	**<0.001**
RT_lag3 * Omission_lag3	-7.38	**<0.001**	-5.08	**<0.001**
RT_lag4 * Omission_lag4	-3.79	**<0.001**	-3.46	**0.001**
RT_lag5 * Omission_lag5	-0.37	0.709	0.46	0.649
RT_Vmax	5.42	**<0.001**	6.34	**<0.001**
RT_Vmax * Trial	2.01	**0.044**	3.63	**<0.001**
Observations	26986		32869	
Marginal R^2 / Conditional R^2	0.228 / 0.363		0.321 / 0.422	

Note. Statistics from a multi-level model predicting participants' choices using the working memory buffer and reinforcement history from the information-compressing RL model. RT_lag1-5 represents the selection buffer, and Omission_lag1-5 (vs. reward as reference condition), the reward buffer, and their interactions reflect assignment of rewards to choices. Model-derived RT_Vmax is the location corresponding to the global value maximum. The lack of a RT_lag5 * Omission_lag5 interaction indicates that the buffer does not extend beyond the four preceding trials.

A significant effect of RT_Vmax indicates that integrated reinforcement history beyond the timeframe of WM buffer influences choices.
